# Microbial response and resistance mechanisms against diverse anthropogenic pollutants

**DOI:** 10.3389/fmicb.2026.1775529

**Published:** 2026-04-14

**Authors:** Ogün Morkoç, Ömer Esen, Alican Topaloğlu, Zeynep Petek Çakar

**Affiliations:** 1 Department of Molecular Biology and Genetics, Istanbul Technical University, Istanbul, Türkiye; 2 Dr. Orhan Öcalgiray Molecular Biology, Biotechnology and Genetics Research Center (ITU-MOBGAM), Istanbul Technical University, Istanbul, Türkiye; 3 Department of Molecular Biotechnology, Turkish-German University, Istanbul, Türkiye

**Keywords:** adaptive laboratory evolution, anthropogenic pollutants, biofilm, evolutionary engineering, omics, oxidative stress, stress resistance, stress response

## Abstract

Anthropogenic activity, driven by industrialization, agricultural practices, and waste disposal, has emerged as a predominant contributing factor to environmental pollution. These activities release substantial amounts of toxic pollutants into the environment, such as heavy metals, organic pollutants, microplastics, and nanomaterials, adversely affecting various ecosystems. These toxic substances can exert considerable stress on various microorganisms, including bacteria, fungi, and microalgae. The impact of anthropogenic pollutants on microorganisms is a nascent area of study, particularly as environmental stressors continue to increase in both quantity and complexity. This review aims to enhance our understanding of how microorganisms (bacteria, microalgae, and fungi) respond to the anthropogenic pollutants including heavy metals, organic pollutants such as polycyclic aromatic hydrocarbons (PAHs), nanomaterials and microplastics. It explores the toxic effects of these pollutants on diverse microbial species. Furthermore, the review covers studies that examine the molecular mechanisms underlying microbial resistance both through natural resistance processes and adaptive laboratory evolution or evolutionary engineering strategies. The review also highlights how omics technologies such as genomics, transcriptomics, proteomics and metabolomics reveal conserved and unique molecular mechanisms to gain insight into the pollutant-specific and organism-specific adaptation strategies. Nevertheless, limitations in community-level multi-omics studies, the relatively limited data on fungi, and the challenges associated with studying mixed cultures hinder a comprehensive understanding of microbial response and resistance mechanisms to anthropogenic pollutants. Addressing these gaps will be pivotal in leveraging the molecular mechanisms to guide the development of novel strategies to obtain pollutant-tolerant strains for bioremediation, bio-monitoring, and synthetic biology applications.

## 1 Introduction

Anthropogenic activities have become a primary source of environmental contamination in recent years. The release of pollutants resulting from these activities has been demonstrated to be toxic to ecosystems, exerting harmful effects on a wide array of organisms, including humans, animals, plants, and microorganisms (Rhind, 2009; Saravanan et al., 2021; Liu L. et al., 2022). Among anthropogenic pollutants, heavy metals are leading contaminants that pose a significant challenge to microbial survival. Even at low concentrations, they exert harmful effects on microbial cells (Dziewit and Drewniak, 2016; Pal et al., 2022). Another class of anthropogenic pollutants are organic pollutants, particularly polycyclic aromatic hydrocarbons (PAHs). They are persistent due to their chemical stability and resistance to degradation (Ufarté et al., 2015). Engineered nanomaterials have been widely applied in fields such as medicine, energy, food, and agriculture (El-Naggar et al., 2024). Their unique physicochemical properties often make them lethal to microorganisms (Zhao et al., 2021; Abishagu et al., 2024). Microplastics have recently emerged as a major environmental concern, affecting a variety of organisms. They can adsorb pollutants and facilitate their dispersion throughout the environment (Zhao X. et al., 2023). These pollutants can notably influence microorganisms by altering their structural integrity, and population dynamics.

**FIGURE 1:**
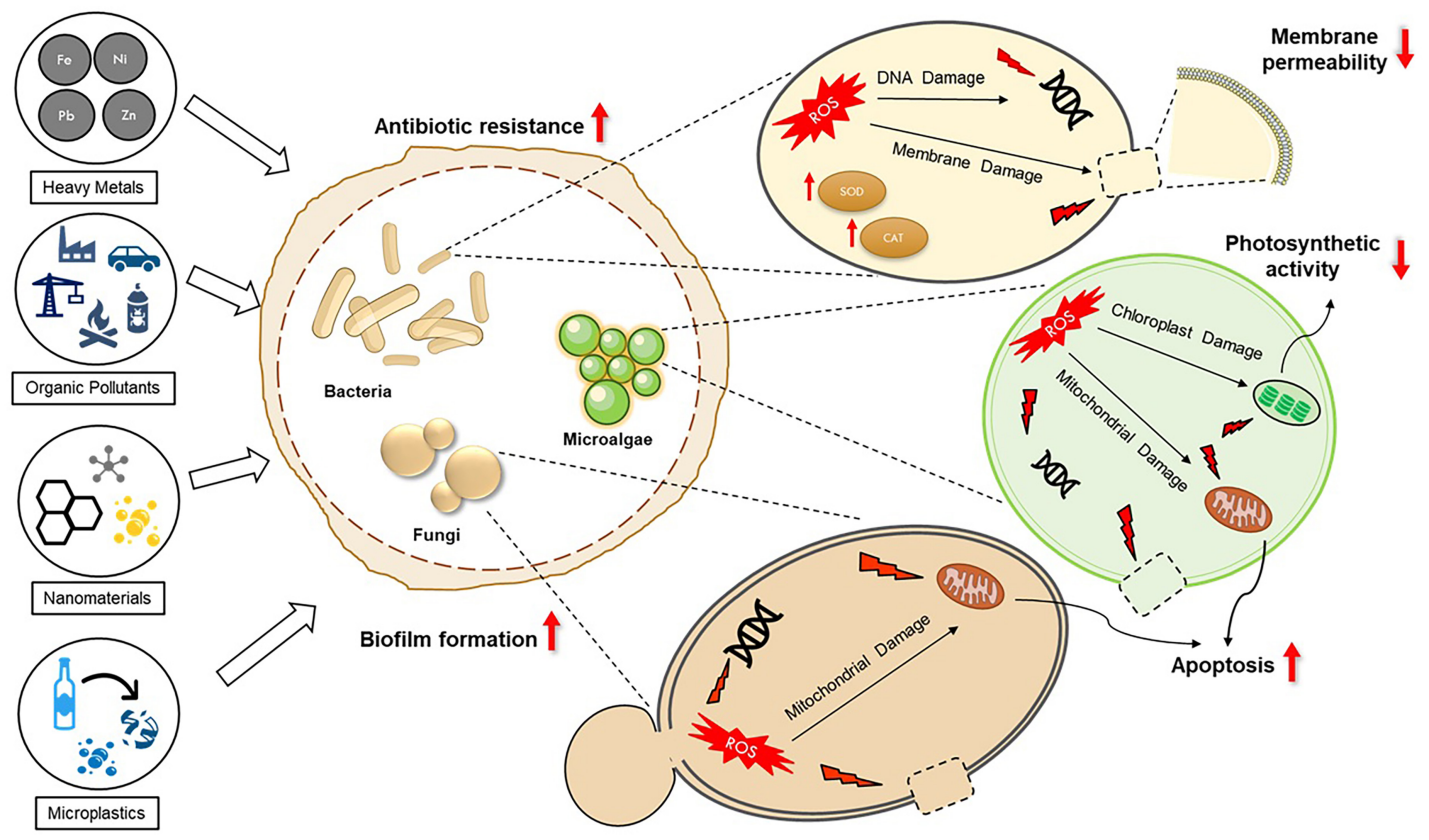
The response and resistance mechanisms of microorganisms (including bacteria, microalgae, and fungi) to various anthropogenic pollutants (heavy metals, organic pollutants, nanomaterials, and microplastics). Pollutants can inhibit growth, induce reactive oxygen species (ROS) production and oxidative stress, increase membrane permeability, and cause DNA damage. Prolonged exposure promotes selective pressure, biofilm formation, and the dissemination of antibiotic resistance genes (ARGs). In eukaryotic microorganisms, pollutant exposure can induce apoptosis, organelle damage, and impair photosynthetic activity in microalgae.

The toxic effects of anthropogenic pollutants have been studied in monocultures of bacteria, fungi, and microalgae using both traditional microbial assays and omics-based approaches (Maurer-Jones et al., 2013; Rihacek et al., 2023; Zheng et al., 2024). The diverse response and resistance mechanisms of microorganisms (bacteria, microalgae, and fungi) to various anthropogenic pollutants are illustrated in Figure 1. Anthropogenic pollutants have been found to inhibit microbial growth, induce the generation of reactive oxygen species (ROS) leading to oxidative stress, increase membrane permeability, and cause DNA damage in microorganisms (Hu et al., 2015; Yang W. et al., 2021; Zhang et al., 2021). Furthermore, anthropogenic pollutants have been demonstrated to induce apoptosis and organelle damage in eukaryotic microorganisms, including microalgae and fungi (Hwang et al., 2012; Yang W. et al., 2021; Kaluç et al., 2024; Zhang Y. et al., 2024). In microalgae, photosynthetic activity is often impaired in response to anthropogenic stressors (Othman et al., 2012; Ansari et al., 2021; Deng et al., 2024). Omics analyses have revealed that exposure to anthropogenic pollutants often alters energy, amino acid, and fatty acid metabolism in microorganisms (Hu et al., 2015; Planchon et al., 2017; Sheng et al., 2023)

The responses of microorganisms to anthropogenic pollutants are of critical importance, not only in terms of pollutant toxicity but also in regulating microbial community dynamics, adaptation to stress, and the emergence and dissemination of resistance genes, including antibiotic resistance genes (ARGs). Long-term exposure to specific anthropogenic pollutants exerts selective pressure on microbial communities, driving the development of tolerance and enabling them to mitigate the adverse effects of these stressors, while acquiring new functional traits (Mishra et al., 2020; Wang et al., 2022; Zhao X. et al., 2023). A key aspect of microbial adaptation is the formation of biofilms, which enhances the accumulation and degradation of pollutants in aquatic ecosystems. Biofilms support the enrichment of specific microbial communities, while simultaneously promoting the selection of resistance traits, including those against diverse pollutants and antibiotics, thereby facilitating the dissemination of resistance genes across environmental and clinically relevant microbial communities (Bakiu et al., 2023; Zarean et al., 2025; Balta et al., 2025). Furthermore, exposure to anthropogenic pollutants can affect microorganism-mediated biogeochemical cycles, including carbon, nitrogen, and phosphorus cycling, by altering the abundance of functional genes involved in these processes (Deng et al., 2022; Yang X. et al., 2021; Mishra et al., 2020; Zhao et al., 2018). Thus, elucidating the mechanisms underlying microbial responses to pollutants is critical for assessing their broader impacts on ecosystems and human health (Zarean et al., 2025).

Omics-based profiling is a powerful approach for relating anthropogenic pollutant pressures in contaminated ecosystems to microbial stress responses, enabling detection of coordinated molecular changes under defined exposure conditions (Wild, 2012). However, the high dimensionality of omics datasets can complicate the interpretation of pollutant-related molecular profiles, as true biological effects are often subtle relative to technical noise and natural variation, thereby increasing the risk of false positives and bias. To minimize these technical challenges, robust study design, adequate replication, and consistent pre-processing are essential (Leek et al., 2010). Integrating genomics, transcriptomics, proteomics, and metabolomics can strengthen mechanistic insight by revealing convergent pathway changes and concordance across omics layers. Nonetheless, such integration can be limited by scale differences, missing data, strain or community variability, and time or growth phase effects; therefore, rigorous and standardized methods are needed to combine multi-omics data reliably (Hasin et al., 2017; Franzosa et al., 2015; Gutleben et al., 2018). Ultimately, connecting molecular changes to microbial resistance and ecosystem-relevant functions requires separating cause from consequence and validating candidate mechanisms within adverse outcome pathway frameworks (Brockmeier et al., 2017). In this way, omics studies provide a vital link between pollutant-driven molecular responses and how anthropogenic pollutants reshape microbial resistance mechanisms and functions.

This review explores the impact of anthropogenic pollutants, including heavy metals, organic pollutants such as polycyclic aromatic hydrocarbons (PAHs), nanomaterials, and microplastics, on microorganisms. Diverse studies investigating the response and resistance mechanisms of bacteria, microalgae, and fungi to these pollutants have been included in this review. Finally, this review also focuses on evolutionary engineering or adaptive laboratory evolution strategies aimed at understanding and enhancing microbial resistance against various anthropogenic pollutants. The literature for this review was identified through a systematic search of Web of Science, PubMed, and Google Scholar databases. Studies that were up to date were prioritized in the selection process. Keywords included combinations of terms related to anthropogenic pollutants (heavy metals, organic pollutants, polycyclic aromatic hydrocarbons (PAHs), nanomaterials and microplastics), toxicity, resistance, omics, and adaptive laboratory evolution–based approaches. Logical operators (AND, OR) were used to refine and broaden the search parameters. Studies were included if they focused on bacteria, fungi, or microalgae. Studies related to biodegradation mechanisms were excluded. To the best of our knowledge, this review provides a novel concurrent examination of microbial responses to diverse anthropogenic pollutants, with a comparative focus on bacteria, microalgae, and fungi.

## 2 Response and resistance mechanisms of microorganisms to heavy metals

Heavy metals are naturally occurring elements with densities over 5 g/cm^3^ and atomic masses exceeding ∼60 amu. The term is often extended to incorporate metallic elements and metalloids, including arsenic (As), tellurium (Te), and selenium (Se), which are persistent, bioaccumulative, and toxic even at trace concentrations (World Health Organization [WHO], 1996; Duffus, 2002; Alotaibi et al., 2021). Heavy metals exert dual roles in living systems; some serve as indispensable micronutrients in trace amounts, whereas others are inherently toxic. Essential trace elements, such as iron (Fe), zinc (Zn), copper (Cu), manganese (Mn), cobalt (Co), and nickel (Ni) function as structural stabilizers and enzyme cofactors in key metabolic pathways (Appenroth, 2010; Kim et al., 2019). In contrast, non-essential elements such as lead (Pb), cadmium (Cd), mercury (Hg), and arsenic (As) have no known physiological function and, owing to the absence of homeostatic mechanisms, can disrupt cellular integrity by damaging membranes, inhibiting enzymes, and inducing genotoxic stress (Loredana Ungureanu and Mustatea, 2022; Nnaji et al., 2024). Although essential metals may become deleterious in excess, their strictly regulated uptake and sequestration mitigate toxicity. Non-essential metals, by contrast, accumulate in an unregulated way, which poses significant risks to the microorganisms and their environment (Bruins et al., 2000; Chandrangsu et al., 2017).

Heavy metal pollution is predominantly driven by anthropogenic sources. Industrial activities such as metal mining and smelting, combustion of fossil fuels, and the use of metal-containing fertilizers and pesticides are acknowledged as primary sources of heavy metal accumulation in soils and aquatic systems (Ali H. et al., 2019; Alengebawy et al., 2021; Angon et al., 2024). Heavy metals such as lead (Pb), copper (Cu), cobalt (Co), zinc (Zn), silver (Ag), cadmium (Cd), and iron (Fe) are toxic environmental contaminants that challenge microbial survival (Pal et al., 2022). Even at low concentrations, heavy metals exert harmful effects on microbial cells. Unlike most organic pollutants, heavy metals are persistent in the environment, as the biological and chemical processes cannot degrade or mineralize them (Mclean and Bledsoe, 1992). Instead, metals undergo redox transformations and ligand-exchange reactions with both inorganic (e.g., hydroxide, sulfide) and organic (e.g., humic acids, siderophores) ligands, thereby altering their speciation, solubility, and transport (Guijarro et al., 2021). Heavy metals circulate among soils, sediments, and waters; however, their mobility and long-term disposition are influenced by environmental factors such as pH, redox potential, organic matter, aeration levels, water content, and temperature, which dictate whether metals are stabilized in solid matrices or remain bioavailable and susceptible to bioaccumulation (Ahemad, 2012).

Heavy metal toxicity in bacteria results from a complex disruption of cellular structure and function. These metals interfere with essential biomolecules via both direct and indirect interactions. One major mode of toxicity is redox-active metals facilitating the generation of ROS, which cause oxidative damage to lipids, nucleic acids, and proteins, thereby disrupting enzymatic activities (Igiri et al., 2018). Heavy metals concurrently alter the structure and function of membrane-bound and cytoplasmic proteins, compromise cell wall integrity, causing morphological deformities, and disturb DNA and RNA synthesis, leading to replication errors and mutagenesis. Moreover, heavy metals can impede the binding of essential cofactors due to strong electrostatic attraction and affinity for analogous molecular sites (Pal et al., 2022). The effects are exacerbated by the ability of certain metals to cause conformational changes in enzymes or to inhibit their activity through competitive or non-competitive interactions with substrates (Gauthier et al., 2014). By binding to cell surfaces and blocking ion channels or transmembrane carriers, they further perturb ion homeostasis (Chen et al., 2014). Collectively, these mechanisms inactivate critical enzymes, disrupt regulatory ion gradients, and interfere with key genetic and metabolic processes, ultimately undermining bacterial viability (Nnaji et al., 2024). In response to the toxic effects of heavy metals, microorganisms have evolved a range of resistance mechanisms. These adaptations include the use of efflux pumps, detoxification enzymes, and metal-binding proteins to mitigate metal toxicity (Pócsi, 2011; Dziewit and Drewniak, 2016; Zhang et al., 2021; Pal et al., 2022).

Meta-omics approaches have improved understanding of microbial responses to heavy metal stress by enabling comprehensive, systems-level characterization of community-wide adaptive mechanisms. Integration of metagenomics, metatranscriptomics, metaproteomics, and metabolomics can overcome the limitations of culture-based and single-omics studies, enabling capture of the full spectrum of genetic potential, gene expression dynamics, protein synthesis, and metabolic fluxes within contaminated environments (Phurailatpam et al., 2022; Khan et al., 2025). Lin et al. (2021) investigated the impact of elevated cadmium, nickel, and chromium levels on microbial communities in denitrifying phosphorus removal sludge (DPRS), using metagenome-assembled genomes (MAGs) and proteomics to map taxon-specific protein responses associated with metal resistance and core nutrient cycling functions. Under metal-induced oxygen limitation, Nitrospira upregulated high-affinity hemoglobin and cytochrome c-type proteins to improve O_2_ uptake, whereas Nitrosomonas increased expression of ammonia monooxygenase and nitrite reductase to maintain nitrogen removal. Dechloromonas, a phosphorus-accumulating organism, preferentially synthesizes polyphosphate for metal detoxification, exhibiting improved energy metabolism, ROS scavenging, and cellular repair mechanisms (Lin et al., 2021). Metagenomic analysis of lake sediments from the Central Andes of Peru illuminated microbial diversity within these ecosystems. 16S rRNA gene amplicon sequencing revealed that heavy-metal contamination in sediments from intensively farmed fish lagoons significantly altered community composition and structure. Cadmium and arsenic-enriched sediments were dominated by Deltaproteobacteria, Actinobacteria, Coriobacteriia, Nitrososphaeria, Acidobacteria, Alphaproteobacteria, Chitinophagia, Nitrospira, and Clostridia, Betaproteobacteria (Custodio et al., 2022). In another study, metagenomic analyses of microbiomes from the rhizosphere of heavy metal-contaminated and mineral-rich environments have identified four keystone groups: Rhizobiaceae, Xanthobacteraceae, Burkholderiaceae, and Actinomycetia which were enriched in genes conferring resistance to heavy metals and other abiotic stresses, facilitating the management of excessive accumulation of zinc/manganese, arsenate/arsenite, chromate, nickel/cobalt, copper, and tellurite. Furthermore, the keystone taxa were observed to utilize both organic and inorganic energy sources, including sulfur, arsenic, and carbon dioxide (Li L. et al., 2023). Shotgun metagenomic and metatranscriptomic sequencing of water samples from the upper oxic layer, chemocline, and deep anoxic layer of the acidic pit lake (*Cueva de la Mora*) revealed depth-dependent shifts in microbial composition that mirror geochemical gradients. Eukaryotes (predominantly Coccomyxa) dominated the oxic zone; Archaea (primarily Thermoplasmatales) prevailed in the anoxic depths; and both bacteria and eukaryotes were abundant at the chemocline. Taxon-specific metal resistance mechanisms included transport (import/export), biochemical transformations, regulation, intracellular accumulation, and extracellular sequestration (Ayala-Muñoz et al., 2020). Herrera-Calderon et al. (2024) isolated bacterial communities from resource islands in the Colombian Caribbean to investigate potential resistance and tolerance to heavy metals. Resistance and tolerance genes were identified in taxonomic groups including Anaerolineales, Acidobacteria, and Proteobacteria. The traits were mediated by various detoxification mechanisms, primarily involving enzymes such as oxidoreductases, metalloproteases, and hydrolases, as well as transmembrane proteins responsible for metal efflux, including efflux pumps and ion transporters (Herrera-Calderon et al., 2024).

Investigating single organisms through laboratory culture is crucial for comprehending the effects of heavy metals, as standardized *in vitro* methods facilitate meticulous control of exposure variables, eliminate environmental confounders, and guarantee reproducibility and comparability in toxicity evaluations (Ma, 2024). In addition, these studies can be performed with pure cultures of bacteria, fungi, and microalgae to gain insight into their bioremediation mechanisms. The utilization of omics approaches is essential for comprehending heavy metal toxicity, as these high-throughput methods reveal molecular profiles, elucidate adverse outcome pathways, and develop novel biomarkers that surpass the capabilities of traditional assays (Rahman et al., 2022; Akash et al., 2023). Recent omics-based studies on bacterial, fungal, and microalgal stress responses to heavy metals, including cadmium (Cd), lead (Pb), copper (Cu), cobalt (Co), zinc (Zn), silver (Ag), and iron (Fe) are summarized in Table 1.

**TABLE 1:** Effects of heavy metals on microorganisms.

Heavy metal pollutant	Concentration	Organism	Observed effects	References
Cadmium (Cd)	1 μg/mL	*Escherichia coli*	Oxidative stress adaptation, reprogrammed energy and storage metabolism, and cross-resistance to chromium, nickel, and cobalt	Wang and Crowley, 2005
Cadmium (Cd)	114.8 μM	*Chlamydomonas reinhardtii*	Reduced growth, upregulated oxidative stress genes, elevated glutathione pathway metabolites	Jamers et al., 2013
Cadmium (Cd)	20 mg/L	*Aspergillus fumigatus*	Metal complexation; increased polysaccharide and protein secretion; elevated intracellular glutathione; activation of energy, amino-acid and glutathione metabolic pathways; ABC transporter upregulation	Tian et al., 2024
Cadmium (Cd) Lead (Pb) Zinc (Zn)	12.5 μg/mL 200 μg/mL 50 μg/mL	*Vibrio cholerae*	Altered membrane properties, efflux activation, and metabolic adaptation for metal detoxification	Zhang et al., 2023
Lead (Pb)	400 ppm	*Proteus mirabilis*	Elevated ROS levels, oxidative stress adaptation, activation of sulfur, nitrogen, and iron metabolism, and metabolic downregulation reflecting a detoxification–energy trade-off	Sevak et al., 2025
Lead (Pb)	128 mg/L	*Lactobacillus plantarum*	Coordinated antioxidant regulation, osmolyte, and amino acid biosynthesis, transporter modulation, and energy conservation	Chen et al., 2025
Lead (Pb)	80 μmol/L	*Chlamydomonas reinhardtii*	Reduced growth and chlorophyll, increased antioxidant enzymes, upregulated ABC transporters, enhanced hormone signaling and antioxidant gene responses	Zheng et al., 2020
Lead (Pb)	500 mg/L	*Pleurotus ostreatus*	ABC transporter upregulation, intracellular chelation/detoxification	Wang et al., 2019c
Copper (Cu)	4 μM	*E. coli*	ROS-independent aggregation of cysteine- and histidine-rich proteins, counteracted by DnaK and trigger factor	(Zuily et al., 2022)
Copper (Cu)	3.2 mM	*Bacillus cereus*	Copper and iron homeostasis regulation, repression of denitrification, and accumulation of metabolites associated with metal transport, membrane remodeling, and antioxidant defense	Wu et al., 2022
Copper (Cu)	12.8 mg/L	*Saccharomyces cerevisiae*	Early oxidative and organic-acid pathway disruption, marked depletion of carboxylic acids, impaired protein synthesis and metabolism, disrupted translation and peptide biosynthetic processes.	Que et al., 2024
Copper (Cu)	3.7 μM	*Ectocarpus siliculosus*	Oxylipin signaling activation, inositol pathway repression, transcription protein regulation, ABC transporter and detox enzymes induction, elevated free fatty acids, autophagy promotion, nitrogen assimilation repression	Ritter et al., 2014
Cobalt (Co)	2.5 mM	*Gluconacetobacter diazotrophicus*	Growth impairment and enhanced iron uptake, modifications in carbohydrate and amino acid metabolism, activation of protein quality control mechanisms, and upregulation of the CzcC efflux system	Pimentel et al., 2025
Cobalt (Co) Iron (Fe)	0.25 mM 2 mM	*Streptococcus suis*	Downregulation of oxidative-stress genes and upregulation of amino-acid ABC transporters, shared cellular perturbations in response to ferrous iron and cobalt	Jia et al., 2020
Cobalt (Co)	80 mg/L	*Rhodopseudomonas palustris*	Upregulation of translation factors, glycolytic enzymes, the phosphate transporter Pst, and methionine adenosyltransferase Mat; enhancement of cobalt bioaccumulation and glutathione-mediated antioxidative defense	Wang et al., 2019b
Cobalt (Co)	4.5 mg/L	*Botryococcus brauni*	Reduced chlorophyll and photosynthesis, increased hydrocarbon production, upregulated fatty acid and hydrocarbon biosynthesis genes, downregulated photosynthesis and carbon metabolism genes	Cheng et al., 2018
Cobalt (Co)	5 mM	*Debaryomyces hanseni*	Upregulation of DNA repair, oxidative stress response, cell wall integrity and growth genes; ROS regulation	Gumá-Cintrón et al., 2015
Silver (Ag)	4 μg/ml	*E. coli*	Perturbation of glycolysis and the oxidative TCA/pyruvate enzymes, glyoxylate-shunt activation, oxidative-defense impairment via Ag^+^ binding and antioxidant-enzyme inactivation	Wang et al., 2019a
Silver (Ag)	6.5 μM	*E. coli*	Inhibition of metabolism, transport, membrane assembly and motility; downregulation of chemotaxis and flagellar genes; upregulation of metal-detoxification genes, divalent-cation transporters, heat-shock proteins, and amino-acid biosynthesis genes; repression of aromatic-compound, sugar and purine catabolic pathways	(Saulou-Bérion et al., 2015)
Iron (Fe)	4 mM	*Bacillus subtilis*	Enhanced acid consumption, proton extrusion via respiratory chain, protein and purine synthesis, cation export, increased polyamine and ammonia production, elevated energy demand, carbon starvation response, oxidative stress adaptation, cross-resistance to chromium, nickel, and cobalt	Yu and Ye, 2016
Iron (Fe)	100 μM	*Phaeodactylum tricornutum*	Enhanced growth and photosynthesis, upregulated Calvin cycle proteins, increased carbon fixation machinery	Zhao et al., 2018
Zinc (Zn)	130 ppm	*Suillus luteus*	Metal exclusion and immobilization, recognition and mitigation of metal induced oxidative stress, transmembrane transport, metal chelation, oxidoreductase activity	Smith et al., 2024

### 2.1 Heavy metals and bacteria

The effects of heavy metals on bacteria have been extensively studied. For example, exposure of *Escherichia coli* K12 strain to cadmium (CdCl_2_) at 1 μg/mL disrupted ribosome assembly, replaced zinc in zinc-binding proteins, and induced a shift to anaerobic metabolism with reduced energy yield. It also activated stress responses, including DNA repair, oxidative and osmotic defenses, acid tolerance, antibiotic resistance, Nudix hydrolases, and metal efflux systems (Wang and Crowley, 2005). In aquatic *Vibrio cholerae* isolates, separate exposures to sublethal Cd^2+^ (12.5 μg/mL), Pb^2+^ (200 μg/mL), and Zn^2+^ (50 μg/mL) elicited a similar cellular response which is characterized by decreased membrane fluidity, increased surface hydrophobicity and inner membrane permeability, along with the coordinated activation of RND and ABC efflux systems, metal-chelating proteins, glutathione peroxidase, extracellular polymeric substance biosynthesis, and energy metabolism pathways, aimed at mitigating metal-induced cytotoxicity (Zhang et al., 2023). Lead exposure (400 ppm) in *Proteus mirabilis* resulted in a 50% increase in ROS, triggering oxidative stress responses, including upregulation of superoxide dismutase (23%) and dehydrogenase (26%), in addition to inducing the expression of genes associated with sulfate and nitrate reduction, iron metabolism, and siderophore production, while downregulating core metabolic functions indicating a trade-off between detoxification and energy expenditure (Sevak et al., 2025). Similarly, Chen et al. (2025) proposed that *Lactobacillus plantarum* exhibits lead (Pb^2+^) resistance via the coordinated regulation of antioxidant defenses, amino acid and osmolyte biosynthesis, transporter activity, and adaptive energy conservation mechanisms. Under anaerobic conditions, exposure to Cu^+^ (4 μM) in *E. coli* led to severe aggregation of cysteine- and histidine-rich proteins, independent of ROS. This effect was alleviated by the cytosolic chaperone DnaK and trigger factor (Zuily et al., 2022). Integrative omics analyses of copper-resistant *Bacillus cereus* T6 have revealed that Cu^+^ exposure induces genes involved in copper transport and iron homeostasis under copper exposure, while denitrification pathways are suppressed, which is accompanied by the accumulation of metabolites linked to metal transporter biosynthesis, membrane remodeling, and antioxidant activity (Wu et al., 2022). Cobalt stress (2.5 mM CoCl_2_) in *Gluconacetobacter diazotrophicus* PAL5 reduced growth by approximately 50% and induced responses such as increased iron uptake, modifications in carbohydrate and amino acid metabolism, activation of protein quality control mechanisms, and upregulation of the CzcC efflux system. In this context, gene knockout studies illustrated the essential role of CzcC in cobalt tolerance (Pimentel et al., 2025). In *Streptococcus suis*, co-treatment with FeSO_4_ (2 mM) and CoSO_4_ (0.25 mM) resulted in the downregulation of oxidative stress genes and the upregulation of amino acid ABC transporters, highlighting the overlapping cellular perturbations caused by ferrous iron and cobalt (Jia et al., 2020). Furthermore, exposure of *Rhodopseudomonas palustris* to 80 mg/L Co^2+^ resulted in the upregulation of translation factors (EF-Tu, EF-NusA, EF-TS), glycolytic enzymes (GAPDH, FBP, METQ, YHFK), the phosphate transporter Pst (12.99-fold), and methionine adenosyltransferase Mat (4.03-fold), thereby enhancing cobalt bioaccumulation and glutathione-mediated antioxidative defense (Wang et al., 2019b). Integrated omics analyses demonstrated that, upon internalization in *E. coli*, Ag^2+^ predominantly interfered with glycolysis and the oxidative branch of the TCA (or pyruvate) cycle by affecting key metabolic enzymes. The metabolic disruption triggered the activation of the glyoxylate shunt as an adaptive mechanism, leading to the impairment of oxidative defense systems due to Ag^2+^ binding and subsequent inactivation of crucial antioxidant enzymes (Wang et al., 2019a). Furthermore, ionic silver exposure inhibited essential growth-related processes, such as metabolism, transport, membrane assembly, and motility, while also downregulating chemotaxis and flagellar genes (*fli*, *flg*, *che*). Simultaneously, it enhanced the expression of metal detoxification genes (*copA*, *cueO*, *zinT*, *ygiW*), divalent cation transporters (*mgtA*, *mntH*, *chaA*, *nhaA*), heat-shock proteins, and amino acid biosynthesis genes (*met*, *arg*), while inhibiting pathways related to the catabolism of aromatic compounds, sugars, and purines (Saulou-Bérion et al., 2015). Excess Fe^3 +^ in *Bacillus subtilis* enhanced acid consumption, proton extrusion through the respiratory chain, and promoted protein and purine synthesis, cation export, and increased production of polyamines and ammonia. These processes elevated energy demand and triggered a carbon starvation response (Yu and Ye, 2016). Overall, bacterial studies demonstrate convergent responses to diverse heavy metals, including the activation of stress response pathways, the employment of metal detoxification and efflux systems, and the disruption of energy metabolism and carbon-related metabolic pathways. However, the balance between detoxification-related protection and growth or energy-yielding processes varies depending on the type of the metal and exposure conditions.

### 2.2 Heavy metals and microalgae

Microalgae employ a stratified defense mechanism to mitigate stress caused by heavy metals. Heavy metals are bound to cell-wall polysaccharides, which restricts their uptake, while membrane transporters are utilized to regulate internal concentrations. Phytochelatins and metallothioneins chelate metals, which are subsequently sequestered in vacuoles or bound to proteins. Simultaneously, ROS from metal stress activate antioxidant enzymes and signaling pathways to facilitate damage repair and restore homeostasis (Suresh Kumar et al., 2015; Tripathi and Poluri, 2021; Xiao et al., 2023a). Exposure of *Chlamydomonas reinhardtii* to cadmium (CdCl_2_) at a concentration of 114.8 μM resulted in reduced cellular growth and upregulation of numerous genes linked with oxidative stress defense mechanisms. This was accompanied by an increased accumulation of metabolites involved in the glutathione biosynthesis pathway (Jamers et al., 2013). Exposure of *C. reinhardtii* to lead Pb(NO_3_)_2_ at 80 μmol/L significantly decreased growth rate and chlorophyll content while increasing antioxidant enzyme activities. It significantly enhanced upregulation of ABC transporters and increased responses associated with hormone signaling and antioxidant-related genes (Zheng et al., 2020). Exposure of *Ectocarpus siliculosus* to copper (CuCl_2_) at 3.7 μM activated oxylipin signaling, repressed inositol pathways, and regulated genes linked to transcription-associated proteins. It also induced ABC transporters, ROS detoxification enzymes (e.g., vanadium-dependent bromoperoxidase), and increased free fatty acids. Moreover, copper stress promoted autophagy and repressed nitrogen assimilation genes (Ritter et al., 2014). Under cobalt stress (4.5 mg/L), *Botryococcus braunii* exhibited reduced chlorophyll content and photosynthetic efficiency, along with an increase in hydrocarbon production. Additionally, genes associated with fatty acid and hydrocarbon biosynthesis were significantly upregulated, whereas those linked to photosynthesis and central carbon metabolism were downregulated (Cheng et al., 2018). In a recent study with *C. reinhardtii*, it was found that the addition of zinc improves copper uptake, resulting in notable accumulation of Cu and Zn at concentrations of 4.21 mg/L Cu and 3.48 mg/L Zn. Superoxide dismutase (SOD) and ascorbate peroxidase (APX) played crucial roles in copper detoxification, whereas zinc facilitated lipid synthesis. The combined Cu-Zn stress redirected carbon flux from starch to lipids, with catalase (CAT) serving as a principal antioxidant enzyme. The upregulation of genes associated with starch and lipid metabolism, as well as energy production, underscores the significance of carbon reallocation in microalgal tolerance to Cu-Zn toxicity (Ye et al., 2023). A study on *Poterioochromonas malhamensis* showed that, despite the internalization of silver nanoparticles (AgNPs), the toxicity is primarily driven by dissolved silver ions released from AgNPs. This toxicity interferes with the metabolic pathways following uptake and accumulation in food vacuoles, causing time-dependent changes in metabolites associated with amino acids, nucleotides, fatty acids, the TCA cycle, antioxidants, photosynthesis, and photorespiration (Liu et al., 2020). Zhao et al. utilized shotgun proteomics in cultures of *Phaeodactylum tricornutum* exposed to different iron concentrations, revealing that increased iron levels markedly improve growth and photosynthetic efficiency. Their main results revealed that excess iron upregulates proteins associated with the Calvin cycle, suggesting an increased abundance of carbon fixation machinery under high-iron conditions (Zhao et al., 2018). Collectively, microalgal studies demonstrate convergent responses to heavy metal exposure, including antioxidant and stress responses, as well as alterations in carbon allocation. In contrast, growth, photosynthetic performance and carbon partitioning toward lipids or hydrocarbons vary, depending on metal identity and microalgal species.

### 2.3 Heavy metals and fungi

Fungi utilize a multicompartmental defense strategy to manage and detoxify heavy-metal stress. This involves the extracellular sequestration of metal ions through binding to cell-wall polysaccharides, the use of intracellular peptide ligands such as metallothioneins and phytochelatins for cytosolic chelation, the transport of metal–ligand complexes into vacuoles for detoxification, and the upregulation of antioxidative pathways to counteract metal-induced ROS. These processes collectively demonstrate their advanced mechanisms for subcellular metal trafficking and oxidative damage mitigation (Kumar and Dwivedi, 2021; Robinson et al., 2021; Jamir et al., 2024). Under cadmium stress, *Aspergillus fumigatus* enhances metal complexation via hydroxyl and carboxyl groups, accompanied by increased secretion of polysaccharides and proteins. Elevated glutathione levels indicated an enhanced redox buffering capacity, while metabolomic analyses revealed the activation of energy, amino acid, and glutathione metabolism pathways involved in damage repair. Additionally, the upregulation of ABC transporters supports intracellular cadmium detoxification through a specific efflux mechanism (Tian et al., 2024). Under lead stress, the white-rot fungus *Pleurotus ostreatus* ISS-1 binds Pb to surface hydroxyl, amide, carboxyl, and sulfhydryl groups, forming PbCO_3_ and PbS. Pb is mainly localized in the cell wall and vacuoles, with ABC transporters likely mediating its transmembrane transport. Oxalic acid and thiol compounds facilitate detoxification via intracellular chelation (Wang et al., 2019c). Under copper stress, the yeast *Saccharomyces cerevisiae* demonstrated an early disruption of oxidative and organic acid metabolic pathways, resulting in a significant decrease in carboxylic acid levels. Protein synthesis and metabolism are compromised, with notable disruptions in translation and peptide biosynthetic processes (Que et al., 2024). Under cobalt stress, the marine yeast *Debaryomyces hansenii* upregulated the genes associated with DNA repair, oxidative stress response, cell wall integrity and growth. The main defense included the activation of non-enzymatic oxidative stress response mechanisms and control of biological production of reactive oxygen species (Gumá-Cintrón et al., 2015). The tolerance of the ectomycorrhizal fungus *Suillus luteus* to zinc correlates with variations in the expression of genes that facilitate metal exclusion and immobilization, alongside those that recognize and mitigate oxidative stress induced by metals. Furthermore, genes with differential expression were identified as being associated with transmembrane transport, metal chelation, oxidoreductase activity, and signal transduction (Smith et al., 2024). Taken together, fungal studies show convergent responses to heavy metal exposure, including metal binding and chelation mechanisms, intracellular sequestration and transport, as well as oxidative stress-related responses. However, downstream metabolic activity and regulation differ depending on the metal type and the fungal taxon: some conditions support coordinated tolerance responses, whereas others lead to the disruption of central metabolic functions and cellular homeostasis.

Across bacteria, microalgae, and fungi, heavy metal exposure is similarly associated with stress response activation and engagement of cellular metal homeostasis/detoxification functions, alongside measurable shifts in metabolic processes that accompany damage mitigation. However, the most prominent features of the response vary with the level of biological organization and physiological processes. In bacteria, multiple studies emphasize on trade-offs in which detoxification, membrane/protein protection, and stress tolerance coincide with suppression or rerouting of energy-yielding metabolism and growth-associated functions under several metals and exposure regimes (Wang and Crowley, 2005; Sevak et al., 2025; Saulou-Bérion et al., 2015; Wang et al., 2019a; Yu and Ye, 2016). In microalgae, heavy metal stress repeatedly intersects with photosynthetic function and carbon allocation, producing metal and species-specific outcomes, including growth and photosynthetic performance, shifts in carbon allocation toward lipids or hydrocarbons (Jamers et al., 2013; Zheng et al., 2020; Cheng et al., 2018; Ye et al., 2023; Zhao et al., 2018). In fungi, the recurring theme is multicompartmental metal management, combining metal interaction/chelation, intracellular handling, transport, and oxidative-stress control; yet the downstream metabolic outcomes differ sharply across metals and species (Kumar and Dwivedi, 2021; Tian et al., 2024; Wang et al., 2019c; Que et al., 2024; Smith et al., 2024). Collectively, these comparisons indicate that while stress signaling, detoxification functions, and metabolic adjustments are common denominators, the balance among detoxification demand, metabolic cost, and physiological performance is distinct across bacterial, fungal, and microalgal systems.

## 3 Effects of organic pollutants on microorganisms

Human activities lead to the release of large amounts of organic compounds into the environment, resulting in widespread environmental and health issues (Naidu et al., 2016). The inherent risk posed by organic pollutants is often attributable to their high toxicity, even at very low levels of exposure (Saha et al., 2017). The most commonly accumulating organic pollutants in the natural environment include polycyclic aromatic hydrocarbons (PAHs), polychlorinated biphenyls (PCBs), and various pesticides. Additionally, dye pollutants, aliphatic compounds from plastics, perfluorinated compounds, phenolic compounds, and organocyanides are also accumulating in the natural environment as a result of anthropogenic activities (Ufarté et al., 2015; Saha et al., 2017). Thus, they are often composed of aromatic rings, planar structures, or polymer chains, which make them chemically stable and resistant to degradation (Ufarté et al., 2015). They are often classified as persistent organic pollutants (POPs), (Jones and de Voogt, 1999) which remain in the environment for long periods and can be transported across various ecosystems on Earth (Ufarté et al., 2015; Alharbi et al., 2018). Organic pollutants originate primarily from industrial, agricultural, medical and municipal activities. In addition to anthropogenic activities, natural events such as volcanic eruptions and wildfires also play a role in introducing these pollutants into the environment. Organic pollutants can bioaccumulate in living organisms, including microorganisms (Welp and Bruk, 1999), potentially impairing biological functions and disrupting ecosystem balance (Alharbi et al., 2018).

Due to their hydrophobic nature (Jones and de Voogt, 1999), organic pollutants can bind to the lipid and protein components of microbial cell membranes (Ren et al., 2018; Mohammed et al., 2020), which cause disorganization of the cell membrane (Ramos et al., 2015). The intake of organic pollutants promotes production of ROS (Ma et al., 2021; Feng et al., 2022). Therefore, organic pollutants have detrimental effects on microorganisms, impacting their cellular structure, membrane permeability, enzymatic activity, and gene expression (Chen et al., 2020; Ma et al., 2021). Organic pollutants can reduce microbial diversity while promoting the enrichment of pollutant-tolerant species in the environment (Cai et al., 2021; Wang et al., 2022, 2024b). Selective pressure can be exerted on microorganisms by pollution and environmental constraints, leading to the selection of those microorganisms that are capable of tolerating and possibly metabolizing the organic pollutants (Miral et al., 2022). The resistance mechanisms of microbial communities under organic pollutant stress are often assessed using molecular approaches such as metagenomics. Wang et al. (2022) investigated the resistance mechanisms of microbial communities in groundwater in China that is contaminated with aromatic hydrocarbons. Their results revealed that at high concentrations of organic pollutants, microbial communities exhibit resistance but show reduced diversity. They observed significant changes in genes associated with metabolism, signal transduction, bacterial transport systems, and cell motility. These findings suggest that the resistance mechanisms of microbial communities involve the regulation of metabolic pathways related to sensing, evading, and excreting toxic compounds (Wang et al., 2022). While various classes of organic pollutants are included in this review, particular emphasis is placed on PAHs as representative model compounds. PAHs have been the focus of extensive research, providing well-characterized mechanistic insights into microbial response and the resistance of organic pollutants. Key findings related to other organic pollutants are summarized in Table 2.

**TABLE 2:** Effects of organic pollutants on microorganisms.

Organic pollutant	Concentration	Organism	Observed effect	References
Phenolic compounds (PNP, PAP, and PhOH)	10 mg/L 50 mg/L	*Escherichia coli* *Acinetobacter* Strain	ROS generation, increased membrane permeability, increase in horizontal gene transfer frequency of ARGs	Ma et al., 2021
Aniline	20 mM	*Rubrivivax benzoatilyticus*	Production of extrapolymeric substance (EPS)	Mohammed et al., 2020
Polyfluoroalkyl substances (PFASs)	0.1 to 200 mg/L	*Pseudomonas stutzeri*	Inhibition of growth, change in cell morphology, ROS generation, PFASs stress altered the expression of genes associated with aerobic nitrification and oxidative stress responses	Qian et al., 2021
Perfluorooctanoic acid (PFOA)	1 mg /L	*E. coli*	Increased membrane permeability to resist PFOA stress, proteomic analysis revealed up-regulation of major catalytic enzymes involved in carbohydrate, lipid and nucleotide metabolism, and the down-regulation of proteins related to energy metabolism and membrane transport activity.	Yang et al., 2017
Polychlorinated biphenyls (PCBs)	0.5–4 μM	*Pseudokirchneriella subcapitata*	Inhibition of growth	Halm-Lemeille et al., 2014
Pesticides (pyrimethanil)	10–110 mg/L	*Saccharomyces cerevisiae*	Inhibition of growth, change in energy and amino acid biosynthesis metabolism, antioxidant response	Gil et al., 2014

### 3.1 Persistent pollutants promote antibiotic resistance

Anthropogenic Pollutants, including PAHs, heavy metals and other persistent organic pollutants, are resistant to degradation and thus persist in the environment, imposing sustained selective pressure on microbial communities. This prolonged exposure can co-select for antibiotic resistance and propagation of antibiotic resistance genes (ARGs). Several studies have demonstrated that antibiotic resistance tends to emerge in areas contaminated with persistent organic pollutants and heavy metals (Sun et al., 2015; Gorovtsov et al., 2018; Maurya et al., 2021; Ding et al., 2021; Miral et al., 2022). Miral et al. (2022) isolated lichen-associated bacterial species from an area in France affected by an oil spill and conducted a functional analysis using a culturomics approach. This method combines culture techniques with mass spectrometry or 16S rRNA sequencing to isolate and taxonomically identify a wide range of microbial diversity. The study revealed that the isolated strains exhibit tolerance to persistent organic pollutants and resistance to various antibiotics (Miral et al., 2022). Two primary mechanisms, co-resistance and cross-resistance, contribute to the co-selection of ARGs. In co-resistance, genes conferring resistance to pollutants and antibiotics are physically linked on the same genetic element. In contrast, cross-resistance occurs when a single resistance mechanism confers tolerance to both antibiotics and pollutants due to shared cellular targets or overlapping cellular response pathways (Rodgers et al., 2019; Zarean et al., 2025). The presence of pollutants in the environment also increases the frequency of horizontal gene transfer rate of ARGs (Lu et al., 2020; Ma et al., 2021). Ma et al. (2021) also demonstrated that under stress from phenolic compounds, the frequency of horizontal gene transfer of ARGs significantly increased between the donor strain *E. coli* HB101 and a recipient Acinetobacter strain isolated from a municipal wastewater treatment process. Biofilm formation has been shown to significantly contribute to the development of tolerance to antibiotics and other stressors, as well as to the transfer of ARGs (Ahmad et al., 2022; Zarean et al., 2025). The extracellular polymeric substance (EPS) matrix traps DNA, facilitates cell-to-cell interactions, and protects bacterial communities from external stressors. Integrons have been demonstrated to enhance the horizontal transfer of ARGs by acquiring and incorporating resistance gene cassettes within biofilm communities (Zarean et al., 2025). A recent study by Chaudhary et al. (2026) demonstrated that the presence of both heavy metals and persistent organic pollutants in wastewater environments in India promotes biofilm formation in Gram-negative bacteria, which indirectly contributes to the spread of antibiotic resistance.

### 3.2 Polycyclic aromatic hydrocarbons (PAHs)

Polycyclic aromatic hydrocarbons (PAHs) are the major class of anthropogenic organic pollutants. They are composed of two or more fused aromatic rings (Sharma et al., 2025). The arrangement of aromatic rings in linear, angular, or clustered forms gives PAHs a wide range of structural configurations. PAHs are generally classified into two groups: low-molecular-weight (LMW) PAHs and high-molecular-weight (HMW) PAHs. The LMW PAH consist of two or three rings, with examples including naphthalene, acenaphthene, acenaphthylene, fluorene, anthracene, and phenanthrene. By contrast, HMW PAHs contain more than three rings, with examples including fluoranthene, pyrene, benzo[a]pyrene, and perylene (Hidalgo et al., 2020). The primary sources of these compounds are anthropogenic activities; however, some are also produced by natural processes. They enter the environment via fossil fuel combustion, industrial activities, oil spills, forest fires, and volcanic activities (Yemele et al., 2024; Sharma et al., 2025). These compounds can become airborne and undergo atmospheric transport (Brown and Brown, 2012; Rodriguez et al., 2015). PAHs are hydrophobic and lipophilic molecules. Because of their hydrophobic nature and poor solubility in water, PAHs are highly resistant to degradation and tend to accumulate in various ecosystems (Stogiannidis and Laane, 2015). For this reason, they are also classified as persistent organic pollutants (Sharma et al., 2025). The lipophilic nature of PAHs allows them to readily adsorb onto particulate organic matter (Bertilsson and Widenfalk, 2002). Thus, they can accumulate in living organisms and have mutagenic and carcinogenic properties, which present a significant threat to both human health and the environment (Saha et al., 2017; Sharma et al., 2025).

Polycyclic aromatic hydrocarbons are toxic to microorganisms. This toxicity has been shown to significantly inhibit metabolic activity and substantially alter the structure of microbial communities (Li et al., 2019; Zhao Z. et al., 2023; Wang et al., 2024c). PAHs exert toxic effects on various microbial communities, including those in soil (Li et al., 2019), airborne environments (Federici et al., 2018), aquatic ecosystems (Bertilsson and Widenfalk, 2002), and even commensal microbes residing on human skin (Sowada et al., 2017). In response to PAHs stress in contaminated soil; microbial diversity, enzyme activity, and the metabolic functions such as carbohydrate metabolism, fatty acid metabolism and amino acid metabolism of soil microorganisms undergo significant changes (Li et al., 2019). Exposure of microbial communities to PAHs has a negative impact on their growth. It was shown that anthracene and phenanthrene PAHs are toxic to aquatic microorganisms and inhibit their growth (Andrushaitis and Platpira, 1991). Furthermore, degradation of PAHs may result in more toxic intermediates. Photochemical degradation, in particular, produces reactive species such as hydroxylated quinones and benzoic acid, which further increase the toxicity of PAHs toward microorganisms (Bertilsson and Widenfalk, 2002; El-Alawi et al., 2002; Ben Othman et al., 2023). The effects of PAHs on microorganisms can also be studied in the laboratory, using pure cultures of bacteria, microalgae and fungi. Selected studies addressing the effects of PAHs on microorganisms are summarized in Table 3.

**TABLE 3:** Effects of polycyclic aromatic hydrocarbons (PAHs) on microorganisms.

Organic pollutant	Concentration	Organism	Observed effects	References
Benzo[a]pyrene, naphthalene, phenanthrene	5 ppb of benzo[a]pyrene 1 ppb of naphthalene 100 ppb phenanthrene	*Escherichia coli*	Genotoxic activity	Kim et al., 2007
Naphthalene, pyrene	10–200 μg/mL	*E. coli*	Inhibition of growth	Wu et al., 2018
12 different PAHs	0.065–4 mg/L	*Vibrio fischeri*	Inhibition of growth, inhibition of luminescence, ROS generation	El-Alawi et al., 2002
Naphthalene	2% w/v in growth medium.	*Rhodococcus erythropolis*	Upregulation of oxidative stress-related genes	Sazykin et al., 2019
Naphthalene	0.5 g/L	*Pseudomonas vesicularis*	Change in the composition and proportion of branched fatty acids to increase membrane fluidity	Mrozik et al., 2004
Naphthalene, phenanthrene	0.1–10 μmol/mg	*E. coli*	Increase in membrane permeability	Sikkema et al., 1994
Naphthalene, phenanthrene, perylene, benzo[a]-pyrene	10–300 μM	*E. coli*	Reduction in metabolic activity, increase in intracellular ROS levels, upregulation of genes related to ROS generation, DNA repair, and efflux pumps	Zheng et al., 2024
Phenanthrene	0.1–10 μg/L	*Chlorella vulgaris Skeletonema costatum*	Inhibition of growth, enhanced SOD activity	Jiang et al., 2022
Anthracene	0.7–5.6 μM	*Chlamydomonas reinhardtii*	Inhibition of growth, decrease in photosynthetic efficiency, increased respiration activity	Aksmann and Tukaj, 2008
Benz(a)anthracene, fluoranthene	0.2–2,000 μg/L	*Nannochloris sp. Picochlorum sp Isochrysis galbana Dunaliella tertiolecta Chaetoceros muelleri Phaeodactylum tricornutum. Alexandrium catenella*	Inhibition of growth, decrease in photosynthetic efficiency	Othman et al., 2012
Naphthalene, pyrene, and benzo(a)pyrene	10–1,000 μg/L	*Nitzschia brevirostris*	Decrease in cell density, membrane integrity, increase in lipid content	Croxton et al., 2015
Benzo[a]pyrene	0.45–36.45 μg/L	*Thalassiosira pseudonana*	Inhibition of growth, upregulation in genes related to oxidative stress and lipid metabolism, expression level changes in proteins involved in lipid metabolism, photosynthesis, and DNA methylation.	Carvalho and Lettieri, 2011; Carvalho et al., 2011
Phenanthrene, pyrene, benzo[a]pyrene	1,000–6,000 mg/L	*Aspergillus nomius* *Trichoderma asperellum*	Inhibition of growth, changes in mycelium pigmentation, and sporulation ability	Zafra et al., 2015
Anthracene, benzo[a]pyrene	140–240 μM	Arbuscular mycorrhizal fungi (*Rhizophagus intraradices (formerly Glomus intraradices)*)	Restriction in spore production, and hyphae length, disruption in membrane lipid content, oxidative stress, inhibition of growth	Debiane et al., 2011; Calonne et al., 2014
benzo[a]pyrene	82.5–330 μM	*Saccharomyces cerevisiae*	Oxidative stress, DNA damage	O’Connor et al., 2013

Furthermore, certain microorganisms, microalgae (Ben Othman et al., 2023), fungi (Padilla-Garfias et al., 2024), and bacteria including genera such as *Pseudomonas* and *Mycobacterium*, have been identified for their ability to degrade PAHs and utilize them as carbon source (Basu et al., 2006). These bacteria developed certain response mechanisms to efficiently degrade PAHs (Mohapatra and Phale, 2021). These mechanisms include chemotaxis (Lacal et al., 2011), regulation of membrane fluidity (Mrozik et al., 2004), and biosurfactant production (Rahman et al., 2002). Understanding the mechanisms of microbial PAHs biodegradation is essential for advancing bioremediation strategies. Comprehensive reviews are available that provide detailed insights into the degradation pathways of PAHs, using omics-based approaches (Gupta et al., 2015; Padilla-Garfias et al., 2024).

#### 3.2.1 PAHs and bacteria

Model organisms such as *E. coli* are often used to study the harmful effects of PAHs (Kim et al., 2007; Wu et al., 2018; Zheng et al., 2024). Even at low concentrations, PAHs inhibit the growth of *E. coli*. Their toxicity also depends on their chemical structure: specifically, PAHs with a greater number of aromatic rings tend to be more toxic (Wu et al., 2018). PAH toxicity also affects bioluminescence: a study with the luminescent bacterium *Vibrio fischeri* showed that exposure to PAHs leads to growth inhibition and reduced luminescence (El-Alawi et al., 2002). Understanding the molecular mechanisms of PAH toxicity is also essential. Kim et al. (2007) used transcriptional analysis to investigate the molecular-level toxicity of various PAHs. They found that, upon PAHs (benzo[a]pyrene and naphthalene) stress, expression levels of the *recA* gene increased. As the expression of rec gene family is related to DNA repair mechanisms, they concluded that these compounds are genotoxic, causing DNA damage in *E. coli* (Kim et al., 2007). The genotoxic effect of PAHs is well-known and it contributes to their carcinogenicity in humans and other animals (Vondráèek and Machala, 2020). As PAH stress damages bacterial DNA, DNA repair mechanism is activated as a cellular response. It has also been reported that exposure of a *Rhodococcus erythropolis* strain to naphthalene induces oxidative stress and leads to the upregulation of oxidative stress-related genes (*sodA*, *sodC*, and *recA)* of cytochrome P450 enzyme. It was suggested that the ROS produced by cytochrome P450 may be a causative agent in the process of DNA damage (Sazykin et al., 2019). These findings are further supported by a study conducted by Zheng et al. (2024), which showed that exposure to small clusters of PAHs induces oxidative stress responses and ROS generation in *E. coli*, regardless of concentration. Transcriptomic analysis under PAHs stress revealed that the genes related to ROS generation, oxidative stress regulation, DNA integration and repair mechanism were upregulated (Zheng et al., 2024). Furthermore, due to the lipophilic nature of PAHs, microbial exposure to PAHs often leads to the loss of membrane integrity and increase in permeability (Sikkema et al., 1995). Sikkema et al. (1994) reported that the accumulation of aromatic hydrocarbons in the *E. coli* membrane leads to decreased activity of cytochrome c oxidase within the membrane. As a result, membrane permeability increases (Sikkema et al., 1994). Intracellular ROS generation is also associated with increased membrane permeability. As membrane permeability rises, more toxic PAHs can enter the cell, resulting in greater toxicity. Upon PAHs stress, genes associated with the efflux system were also upregulated in *E. coli* to expel PAH molecules (Zheng et al., 2024). Ultimately, studies conducted on bacteria to investigate the effects of PAHs have demonstrated that PAH toxicity induces oxidative stress, leading to DNA damage and loss of membrane integrity. However, the extent of toxicity depends on the chemical structure of the PAHs. In response to this stress, bacteria activate oxidative stress response pathways, DNA repair systems, and efflux mechanisms.

#### 3.2.2 PAHs and microalgae

Effect of PAHs on other microorganisms such as microalgae have also been studied extensively. Microalgae play a crucial role in research related to the organic pollution of water, as they are naturally present in aquatic environments (Ben Othman et al., 2023). The response of microalgae to PAH exposure is mainly correlated with that of bacteria. Single culture studies with exposure of microalgae to PAHs revealed that the PAHs toxicity leads to growth inhibition (Aksmann and Tukaj, 2008; Jiang et al., 2022), impaired membrane structure (Croxton et al., 2015), oxidative stress (Jiang et al., 2022), and reduced photosynthetic efficiency (Othman et al., 2012; Ben Othman et al., 2023). The toxic effects of PAHs on microalgae depend on the cell size and lipid content: smaller microalgae tend to uptake more pollutants and are more sensitive to PAHs toxicity (Othman et al., 2012). Jiang et al. (2022) demonstrated that under phenanthrene stress, the growth and photosynthetic performance of the green microalga *Chlorella vulgaris* and the diatom *Skeletonema costatum* were inhibited. Both species showed increased superoxide dismutase (SOD) activity, while *C. vulgaris* also exhibited elevated catalase (CAT) activity (Jiang et al., 2022). The increased activity of SOD and CAT indicates the activation of the antioxidant defense mechanism in response to the harmful effects of ROS (Sigaud-Kutner et al., 2002). Conformational changes in membrane structure may contribute to the decrease in photosynthetic efficiency. This reduction creates an increased energy demand. Aksmann and Tukaj observed that, upon exposure PAHs, stimulation of respiration was induced as a compensatory response in *C. reinhardtii* (Aksmann and Tukaj, 2008). In another study, PAHs-exposed cultures of the diatom *Nitzschia brevirostris* showed an increase in lipid content, which was associated with the compensation for the elevated energy demand (Croxton et al., 2015). The decrease in photosynthetic efficiency in microalgae due to PAHs contamination poses a significant threat to atmospheric carbon dioxide uptake, as microalgae are key primary photosynthetic organisms (Carvalho and Lettieri, 2011). Furthermore, transcriptomic analysis of the marine diatom *Thalassiosira pseudonana* revealed that benzo(a)pyrene exposure leads to the upregulation of genes associated with oxidative stress, general stress responses, and lipid metabolism. These findings suggest that, in response to PAHs stress, microalgae activate general stress response mechanisms and alter lipid metabolism (Carvalho et al., 2011). Quantitative proteomic analysis further supported these findings, showing that exposure to benzo(a)pyrene alters the expression of proteins involved in lipid metabolism and photosynthesis of *T. pseudonana* (Carvalho and Lettieri, 2011). Overall, the response and resistance mechanisms of microalgae to PAHs toxicity show patterns similar to those observed in bacteria, including the activation of oxidative stress responses and alteration of lipid metabolism. Responses to PAHs differ based on the microalgal species and the specific characteristics of the compounds. Beyond their effects on bacteria, PAHs can disrupt microalgal photosynthetic activity and elevate energy requirements.

#### 3.2.3 PAHs and fungi

Polycyclic aromatic hydrocarbons also have a detrimental effect on the fungi. Upon PAHs stress, fungal strains exhibit growth impairment, decrease in sporulation, and alteration in membrane structure (Debiane et al., 2011; Calonne et al., 2014; Zafra et al., 2015). A study conducted on *Aspergillus nomius* and *Trichoderma asperellum* revealed that increasing doses of PAHs stress lead to growth inhibition, morphological changes such as altered mycelium pigmentation, and suppression of sporulation (Zafra et al., 2015). It was suggested that the change in pigmentation may be linked to alterations in membrane permeability caused by PAHs stress. Findings from studies on *Rhizophagus intraradices* (formerly named *Glomus intraradices* (Walker et al., 2021) indicate that PAHs exposure disrupts membrane lipid composition, and induces oxidative stress (Debiane et al., 2011; Calonne et al., 2014). The interaction between PAHs and fungal lipid membranes was investigated by Wójcik et al. (2022), using a plasma membrane model. It was found that PAHs can alter the order of the lipid bilayer and affect the function of lipids in the plasma membrane (Wójcik et al., 2022). Most studies on the effects of PAHs on fungi have focused on their biodegradation capabilities, as research in PAHs-contaminated areas has shown that fungi are present across diverse ecosystems (Aranda, 2016). These fungi incorporate PAHs into their plasma membranes for oxidation and further metabolism (Müncnerová and Augustin, 1994; Padilla-Garfias et al., 2024). Thus, they have developed a resistance mechanism against the toxic effects of these pollutants (Aranda et al., 2013). To understand the mechanisms of toxicity and cellular resistance to PAHs, O’Connor et al. conducted genomic, transcriptomic, and proteomic analyses of the yeast *S. cerevisiae* exposed to benzo[a]pyrene. Gene ontology analysis revealed that PAHs toxicity in yeast is associated with oxidative stress, DNA damage and repair, drug transmembrane transport, and detoxification through export (O’Connor et al., 2013). In conclusion, studies on fungi have revealed that conserved stress responses, including those related to oxidative stress and membrane remodeling, are prevalent. In contrast to bacteria and microalgae, fungi display distinct morphological responses to PAHs stress, including the suppression of sporulation.

In general, exposure to organic pollutants, particularly PAHs, induces oxidative stress and disrupts membrane permeability in bacteria, microalgae, and fungi, ultimately resulting in growth inhibition. In contrast to bacteria, organic pollutant toxicity in microalgae is also associated with reduced photosynthetic activity (Othman et al., 2012; Ben Othman et al., 2023), whereas in fungi it manifests as distinct morphological changes such as reduction in sporulation (Zafra et al., 2015). In response to organic pollutant stress, microorganisms activate oxidative stress response pathways, increase their energy demands, and may subsequently acquire the ability to degrade these toxic compounds (Mohapatra and Phale, 2021). However, as microorganisms in ecosystems develop resistance to organic pollutants, they may concurrently acquire antibiotic resistance through mechanisms such as biofilm formation and horizontal gene transfer, thereby posing significant ecological risks (Zarean et al., 2025).

## 4 Microbial response and resistance to nanomaterials

Nanomaterials are often defined as materials with particle sizes ranging from 1 to 100 nm. They consist of unbound particles or exist in aggregated or agglomerated forms (Bleeker et al., 2013). Nanomaterials can be organic, inorganic, or organometallic materials that exhibit size- and shape-dependent variations in their chemical, physical, or electrical properties (Hochella et al., 2019). Natural nanomaterials are part of the Earth’s system and are formed through natural biogeochemical or mechanical processes without any anthropogenic influence. In contrast, nanomaterials can also be engineered to possess novel, tunable properties, offering significant advantages for industrial applications (Hochella et al., 2019; Lespes et al., 2020). Engineered nanomaterials, such as metallic nanoparticles, polymeric nanoparticles, quantum dots, nanotubes, and nanowires have been applied in a wide range of areas including medicine, energy, food, cosmetics and agriculture (El-Naggar et al., 2024). Unlike natural nanoparticles, engineered nanoparticles do not aggregate to form larger structures; therefore, they tend to persist longer and bioaccumulate in the environment (Handy et al., 2008). The release of engineered nanoparticles into the environment is steadily increasing due to intensified human activities (Gottschalk and Nowack, 2011; Giese et al., 2018). The presence of engineered nanoparticles, alongside natural ones, is likely to have a negative impact on both ecosystems (Maurer-Jones et al., 2013) and human health (Oberdörster et al., 2005; Liu Y. et al., 2022).

The unique properties of nanomaterials, such as their high surface area and extraordinarily small size, make them bioactive molecules (Yan et al., 2017; Zhao et al., 2021). They can interact with various biomolecules, including lipids, peptides, and nucleic acids (Lespes et al., 2020). The toxicity of nanomaterials has raised significant concern in recent years (Garner and Keller, 2014; Abishagu et al., 2024). Nanomaterials can exert harmful effects on human tissues and cell culture. They are capable of circulating throughout the body and accumulating in various tissues, thereby inducing elevated oxidative stress, mitochondrial perturbation, and DNA damage which may result in cell death (Oberdörster et al., 2005; Liu Y. et al., 2022). Thus, a new and distinct field of research, known as “nanotoxicology” has emerged to study the effects of nanomaterials and nanoparticles on living organisms. The toxicity mechanisms of nanomaterials are influenced by factors such as their size, shape, chemical composition, surface characteristics, and their state of agglomeration or aggregation (Liu Y. et al., 2022). As particle size decreases, the surface area-to-volume ratio increases, resulting in a larger proportion of atoms or molecules being exposed on the surface. Specific surface groups on nanomaterials can serve as reactive sites, influencing their chemical behavior. Such size-dependent changes in the physicochemical and structural properties of engineered nanomaterials are thought to play a key role in their interactions with biological systems and may contribute to their potential toxicological effect (Nel et al., 2006).

Interactions between nanomaterials and microorganisms has been studied at both the microbial community and the single-organism level (Yang X. et al., 2021; Yamini et al., 2023). Due to their unique physicochemical properties, nanomaterials can adhere or be absorbed by microorganisms and change their cellular homeostasis. Nanomaterials can affect various microbial communities in soil, and aquatic environments. Their interaction with microorganisms results in the production of ROS, which can have detrimental effects on the cell membrane, DNA, and the protein structure (Yang X. et al., 2021; Yamini et al., 2023; Abishagu et al., 2024). Long-term exposure to nanomaterials can alter microbial composition and diversity (Mishra et al., 2020; Yang X. et al., 2021). Yang X. et al. (2021) demonstrated that chronic exposure to carbon nanotubes (CBNs) in microbial communities at wastewater treatment sites led to increased ROS production and changes in the expression of several genes and enzyme functions involved in microbial nitrogen metabolism. Such alterations in microbial metabolic activity may disrupt microbe-driven elemental cycles (such as carbon, nitrogen, and phosphorus) (Yang X. et al., 2021). For example, it was shown that exposure to silver nanoparticles (AgNPs) leads to a reduction in the abundance of functional genes involved in the nitrogen and phosphorus cycles within soil bacterial communities (Mishra et al., 2020). Another study conducted on nitrifying bacteria, such as *Nitrosomonas europaea*, *Nitrosospira multiformis*, and *Nitrosococcus oceani*, revealed that treatment with AgNPs inhibits their nitrification potential (Beddow et al., 2014).

Apart from their direct toxicity, nanomaterials can interact with organic pollutants, either mitigating their toxic effects or altering their bioavailability (Dinesh et al., 2012). It is well-known that nanomaterials exert antimicrobial effects on microorganisms (Hussain et al., 2023; Wei et al., 2023). Due to their antimicrobial properties, nanomaterials have been used as either antimicrobial agents or antimicrobial drug carriers (Beyth et al., 2015; Patra et al., 2018). Thus, they are classified as non-antibiotic antibacterial agents, a categorization that may present a new challenge in the context of antibacterial resistance (Raza et al., 2021). Their antibacterial properties make them desirable for various applications, including pest management and the control of plant microbiomes, and their use as pesticides and fungicides. However, their toxicity also affects beneficial microorganisms, posing an environmental risk. Consequently, understanding the cellular-level toxicity of engineered nanomaterials on microorganisms is crucial for developing more effective applications (Yamini et al., 2023; Abishagu et al., 2024). The toxic effects of nanomaterials have been studied in bacteria, fungi and microalgae, using analysis of growth, morphological changes and ROS generation. In addition, omics approaches are utilized to elucidate molecular mechanisms of nanomaterial toxicity (Maurer-Jones et al., 2013; Hu et al., 2015; Masaki et al., 2015; Rihacek et al., 2024; Khosrovyan et al., 2025). Table 4 provides a summary of key studies published in recent years.

**TABLE 4:** Effects of nanoparticles (NPs) on microorganisms.

NPs type	Size	Shape	Concentration	Organism	Observed effects	References
ZnO, TiO_2_ CuO, Co_3_O_4_	47–106 nm for ZnO, 17–64 nm for TiO_2_, 17–45 nm for CuO, and 51–132 nm for Co_3_O_4_	Spherical, rhomboid, rod, quadrate	0.01–5 ppm for ZnO, 1–750 ppm for TiO_2_, 0.1–7.5 ppm for CuO, and 40–80 ppm for Co_3_O_4_	*Escherichia coli*	Decreased viability, induced ROS production	Dasari et al., 2013
ZnO, TiO_2_	42–71 nm	Spherical	0.05 ppm for ZnO, 2.5 ppm for TiO_2_	*E. coli*	Induced ROS production, alterations in glycine, serine, and threonine metabolism	Pathakoti et al., 2020
TiO_2_	8.5–37 nm	Spherical, rod, cubes	10–100 ppm	*E. coli*	Alterations in energy metabolism, growth, and amino acid metabolism.	Planchon et al., 2017
MoS_2_	*Nd	Nanosheets	1–1,000 μg/mL	*E. coli*	Induced ROS production, cell shrinkage, damaged cellular structure, changes in amino acid metabolism	Wu et al., 2016
Graphene	Size < 300 nm, thickness < 1 nm	Nanosheets	400 μg/mL	*E. coli*	Decreased viability, antibacterial effect, damaged cellular structure, induced ROS production	Liu et al., 2011
CuO	<50 nm	*Nd	50–250 mg/L	*Desulfovibrio vulgaris*	Inhibition of growth, induced ROS production, downregulation of the genes related to cell motility	Chen et al., 2019
AgNPs	20–90 nm	Spherical, rod	184–350 μg/mL	*E. coli, Staphylococcus aureus, Bacillus subtilis, Pseudomonas aeruginosa, Klebsiella pneumoniae*	Inhibition of growth, antibacterial effect, cell wall damage	Acharya et al., 2018
AgNPs	10–50 nm	Spherical	10 mg/L	*Azotobacter vinelandii*	Inhibition of growth, cell injury, oxidative stress	Zhang et al., 2018
Nanosized zero valent iron (nZVI)	<50 nm	*Nd	1–10 g/L	*Bacillus cereus*	Decreased viability, entry in early sporulation stage, repression of proteins involved in cell motility and cell wall biosynthesis, upregulation of proteins involved in oxidative stress response	Fajardo et al., 2013
Ag, ZnO, CuO, Al_2_O_3_, and TiO_2_	12.6–58 nm	*Nd	62.5–1,500 μg/mL	*Azotobacter chroococcum, Bacillus thuringiensis, Pseudomonas mosselii, and Sinorhizobium meliloti*	Increased membrane permeability, biofilm formation, cellular damage	Ahmed et al., 2020
AgNPs	17–40 nm	*Nd	0.5–50 mg/L	*Nitrosomonas europaea*, *Nitrosospira multiformis*, and *Nitrosococcus oceani*	Decrease in nitrification	Beddow et al., 2014
TiO_2_	19.9–68.7 nm	Spherical and cuboid	5–500 μg/L	Marine cyanobacteria *Prochlorococcus*	Inhibition of growth by direct physical effects: entrapment of microbial cells upon aggregation of TiO_2_ NPs.	Dedman et al., 2021
Graphene oxide (GO), carboxyl single-walled carbon nanotubes (C-SWCNT)	0.8–1.2 nm for GO, 1–1.6 nm for C-SWCNTs	Nanosheet, nanotube	0.02–10 mg/L	*Chlorella vulgaris*	Decrease in cell viability, increase in plasmolysis, loss in mitochondrial membrane potential, increase in ROS levels, alterations in carbohydrate, amino acid, fatty acid, and urea metabolic pathways	Hu et al., 2015
ZnS quantum dots (QDs)	1.1–2 nm	Crystalline structure	20–100 mg/L	*C. vulgaris*	Decrease in viability, photosynthetic activity and mitochondrial activity, increase in ROS production, cell wall damage	Deng et al., 2024
TiO_2_, CeO_2_	TiO_2_ 5–10 nm, CeO_2_ 10–30 nm	*Nd	2.5–40 mg/L	*Phaeodactylum tricornutum*	Inhibition of growth, cellular damage, decrease in photosynthetic activity, increase in ROS production	Deng et al., 2017
AgNPs	20 nm	*Nd	0.01–4 mg/L	*P. tricornutum*	Inhibition of growth, and apoptosis, change in cell morphology, increase in ROS production, loss of membrane integrity, change in mitochondrial membrane potential	Zhang Y. et al., 2024
SiO_2,_ ZnO	*Nd	*Nd	56.80 mg/L for ZnO and 14 mg/L for SiO_2_	*Chlamydomonas* sp.	Inhibition of growth, DNA damage, reduced photosynthetic activity, upregulation of the genes related to apoptosis and carbohydrate biosynthesis	Ghariani et al., 2025
AgNPs	15 nm	*Nd	2–10 mg/L	*Fusarium solani*	Antifungal effect, inhibition of growth, increase in ROS production, cellular damage, apoptosis induction	Shen et al., 2020
CeO_2_	<10 nm	*Nd	10–250 ppm	*Saccharomyces cerevisiae*	Decrease in oxygen uptake, alteration in released organic metabolites after exposure	Masaki et al., 2015
Cadmium sulfide (CdS) Quantum dots	5–50 nm	*Nd	1–100 mg /L, 0–250 mg/L	*S. cerevisiae*	Decrease in yeast viability, inhibition of spore development, mitochondrial damage	Marmiroli et al., 2016, 2023; Rossi et al., 2022
Cadmium telluride (CdTe) Quantum dots	2.3 nm	Spherical	6–60 nM	*S. cerevisiae*	Decrease in viability, disruption of membrane integrity, induction of autophagy, disturbance of metabolic processes	Wei et al., 2022
Carbon nanotubes	10–35 nm	*Nd	160–800 mg/L	*S. cerevisiae*	Decrease in viability, increase in ROS production	Martín et al., 2021
Mn_2_O_3_	30–60 nm	Spherical	100–1,000 mg/L	*S. cerevisiae*	Membrane damage, inhibition of O_2_ consumption	Otero-González et al., 2013
AgNPs	10–80 nm	Spherical	0.01–100 mg/L	*S. cerevisiae*	Decrease in cell viability, cell wall and membrane damage, increased vacuole size	Kasemets et al., 2019
AgNPs	10–30 nm	Spherical	5–80 μg/L	*Candida albicans*	Antifungal effect, ROS generation, membrane damage, change in fatty acid concentration	Radhakrishnan et al., 2018
AgNPs	3 nm	Spherical	2 μg/L	*C. albicans*	Antifungal effect, ROS generation, decrease in mitochondrial membrane potential, apoptosis induction	Hwang et al., 2012

*Nd, not determined.

Studies have shown that some microorganisms, especially bacteria, have evolved to gain resistance against nanomaterials (Wu et al., 2010; Siemer et al., 2019; Valentin et al., 2020). To protect themselves from oxidative stress caused by nanomaterials, microorganisms often induce the expression of SOD and CAT enzymes (Martín et al., 2021; Deng et al., 2024; Rihacek et al., 2024). Nanoparticles usually inhibit biofilm formation. However, studies have shown that by upregulating enzymes involved in extracellular matrix formation, microorganisms can induce biofilm development to overcome the toxic effects of nanomaterials (Yang and Alvarez, 2015; Auger et al., 2019; Ahmed et al., 2020). Other mechanisms of resistance involve the activation of efflux pumps (Yang et al., 2012), alterations in cellular morphology (Hachicho et al., 2014), and metabolic responses (Niño-Martínez et al., 2019). Furthermore, studies have indicated that resistance to nanomaterials can lead to the development of cross-resistance to antibiotics (Yang et al., 2012) and heavy metals (Guo et al., 2017; Valentin et al., 2020).

### 4.1 Nanomaterials and bacteria

The toxicity of nanomaterials depends on their concentration and physical properties. The toxicological impact of nanoparticles increases as their particle size decreases and surface area increases (Masaki et al., 2015; Ghariani et al., 2025). Exposure to nanoparticles generally resulted in growth impairment, oxidative stress response, and membrane disruption in microorganisms (Acharya et al., 2018; Zhang et al., 2018; Zhang Y. et al., 2024; Ahmed et al., 2020). Consequently, the damage to the cell membrane results in the leakage of cellular contents, which ultimately leads to cell death (Hu et al., 2015; Zhang et al., 2018, 2021). Studies on the model organism *E. coli* involving metallic nanoparticles showed that the attachment of dissolved metal ions to the cell surface leads to the formation of hydroxyl radicals, thereby promoting ROS generation (Dasari et al., 2013; Pathakoti et al., 2020). A higher concentration of ROS causes oxidative stress, resulting in damage to membrane lipids, proteins, and DNA (Ševcù et al., 2011). Analyses conducted using scanning electron microscopy (SEM) and transmission electron microscopy (TEM) have revealed that nanoparticles can physically damage the cell wall and the membrane, leading to the loss of membrane permeability (Liu et al., 2011; Wu et al., 2016; Deng et al., 2017; Ahmed et al., 2020; Dedman et al., 2021; Zhang Y. et al., 2024). The shape of the nanomaterial also contributes to the extent of damage; spherical nanoparticles tend to cause more harm than one-dimensional nanostructures such as nanowires and nanorods (Acharya et al., 2018). The nanosheets can also damage the cellular membrane due to their sharp edges (Liu et al., 2011). Omics analyses have shown that nanoparticle exposure often leads to metabolic alterations, including changes in carbohydrate, energy and amino acid metabolism (Wu et al., 2016; Planchon et al., 2017; Pathakoti et al., 2020; Rihacek et al., 2024). It was observed that an increase in glycine levels may help scavenge ROS and protect *E. coli* cells from oxidative stress (Pathakoti et al., 2020). Metabolomic analyses also revealed elevated levels of putrescine, a polyamine that plays an important role in ion scavenging (Planchon et al., 2017; Pathakoti et al., 2020). Proteomic analyses showed that upon exposure to titanium dioxide and zinc oxide nanoparticles, proteins involved in energy, carbohydrate biosynthesis and metabolism, oxidative stress response, and electron transport chain were upregulated in *E. coli* (Planchon et al., 2017; Rihacek et al., 2024). The multi-omics study conducted by Rihacek et al. revealed that, after exposure to ZnO nanoparticles, the expression of enzymes involved in the TCA cycle and the synthesis of carbohydrates, including trehalose, was upregulated in *E. coli* (Rihacek et al., 2024). It was also shown that the exposure to nanomaterials can affect cell motility in various bacteria, such as the sulfate-reducing bacterium *Desulfovibrio vulgaris* (Chen et al., 2019) and the soil bacterium *B. cereus* (Fajardo et al., 2013). Omics analyses revealed that the genes and proteins associated with cell motility were downregulated in *B. cereus* and *D. vulgaris*, upon exposure to nanosized zero-valent iron particles and copper oxide (CuO) nanoparticles, respectively (Fajardo et al., 2013; Chen et al., 2019). These metabolic alterations reflect the bacterial stress response to nanomaterials, enabling the diversion of metabolic flux toward the production of energy needed for protection and adaptation. Taken together, studies on bacteria exposed to nanoparticles indicate that nanomaterial toxicity can induce oxidative stress and impair membrane integrity. In response, bacteria modulate their carbon and energy metabolism and activate oxidative stress response pathways. The exact mechanisms may vary depending on the physicochemical properties of the nanoparticles and the bacterial species.

### 4.2 Nanomaterials and microalgae

Studies conducted on microalgae have also shown that the primary mechanism of nanomaterial toxicity arises from oxidative stress induced by ROS production (Hu et al., 2015; Deng et al., 2017, 2024; Zhang Y. et al., 2024). An increase in ROS levels leads to the loss of membrane integrity, DNA damage, and impaired mitochondrial and chloroplast activity in microalgae (Deng et al., 2024; Zhang Y. et al., 2024). Similar to findings in bacteria, adherence of nanomaterials on cell surface physically damages the cell structure and results in oxidative stress (Lei et al., 2016; Dedman et al., 2021). Exposure to metallic nanoparticles and quantum dots has also been shown to inhibit the photosynthetic activity of microalgae (Deng et al., 2017, 2024; Ghariani et al., 2025). Oxidative stress induced by nanomaterial exposure leads to a reduction in chlorophyll *a* and *b* production, thereby impairing photosynthesis (Liang et al., 2020; Ghariani et al., 2025). Studies have also shown that exposure to nanomaterials triggers apoptosis in microalgae. Transcriptomic analysis of *Chlamydomonas sp*. exposed to silicon dioxide (SiO_2_) and ZnO nanoparticles revealed that the genes related to apoptosis were overexpressed (Ghariani et al., 2025). Zhang Y. et al. (2024) demonstrated that exposure to AgNPs increased the number of apoptotic cells in the diatom *P. tricornutum*. It was found that apoptosis was initiated by mitochondrial damage (Zhang Y. et al., 2024). Mitochondrial structural damage and increased membrane permeability were also observed in other studies conducted with *Chlorella vulgaris* exposed to various nanomaterials (Hu et al., 2015; Deng et al., 2024). Furthermore, metabolomic analyses by Hu et al. (2015) revealed that nanoparticle exposure leads to alterations in the amino acid metabolism of microalgae, consistent with the observations in bacteria. Ultimately, the molecular mechanisms underlying responses of microalgae to nanomaterials are similar to those observed in bacteria. However, in contrast to bacteria, exposure of microalgae to nanomaterials can damage mitochondria and chloroplasts, leading to impaired photosynthesis and the induction of apoptosis.

### 4.3 Nanomaterials and fungi

The response to nanomaterials has also been investigated in another eukaryotic kingdom: fungi. Compared to bacteria, fungi exhibit lower sensitivity to most common nanoparticles, owing to their rigid cell wall (Kasemets et al., 2019). Nonetheless, nanoparticles have demonstrated antifungal activity against pathogenic fungi species, including *Candida albicans* and *Fusarium solani* (Hwang et al., 2012; Radhakrishnan et al., 2018; Shen et al., 2020). Studies conducted with fungi revealed that nanomaterial exposure leads to growth inhibition (Shen et al., 2020; Rossi et al., 2022; Wei et al., 2022), membrane damage (Otero-González et al., 2013; Kasemets et al., 2019; Wei et al., 2022), oxidative stress (Hwang et al., 2012; Radhakrishnan et al., 2018; Shen et al., 2020; Martín et al., 2021), inhibition of spore development (Rossi et al., 2022), DNA damage, and apoptosis induction (Hwang et al., 2012; Radhakrishnan et al., 2018; Shen et al., 2020). Similar to microalgae, omics analyses have revealed that nanomaterial exposure results in mitochondrial dysfunction (Marmiroli et al., 2016; Pagano et al., 2018; Peng et al., 2018). Mitochondrial disintegration results in cytochrome c release and induction of apoptosis. *C. albicans* cells exposed to AgNPs showed increased ROS production, resulting in mitochondrial dysfunction and apoptotic features (Hwang et al., 2012). In addition, Wei et al. (2022) reported that exposure to cadmium telluride (CdTe) quantum dots induces autophagy in *S. cerevisiae*. The autophagy pathway was upregulated, according to their transcriptomic analysis results. In another study, Kasemets et al. (2019) showed that exposure to AgNPs in *S. cerevisiae* results in changes in surface morphology and an increase in vacuole size. A toxicogenomic study conducted by Marmiroli et al. (2016) also showed that the genes associated with tolerance to cadmium sulfide (CdS) quantum dots in mutant *S. cerevisiae* strains, (e.g., *VAC7*, *VTC2*, and *VMA4)* are related to vacuolar processes. Thus, the enlargement of vacuoles and induction of autophagy may indicate the development of resistance in *S. cerevisiae* to AgNP-induced stress (Kasemets et al., 2019; Wei et al., 2022). Furthermore, a transcriptomic study on the soil fungus *F. solani* following AgNP exposure revealed that the genes related to carbohydrate and energy metabolism were upregulated, while genes related to the cell cycle were downregulated, indicating an increased energy demand following nanomaterial exposure (Shen et al., 2020). Collectively, the responses of fungi to nanomaterials largely converge with those observed in bacteria and microalgae, except for distinct morphological changes and the induction of apoptosis and autophagy. Fungi are generally more resilient than other microorganisms, possibly due to their rigid cell wall. However, studies on fungi remain limited compared to those on bacteria and microalgae, highlighting the need for further research to elucidate their response and resistance mechanisms to nanomaterials.

Across bacteria, microalgae, and fungi, nanomaterial exposure induces responses comparable to those caused by other pollutants, particularly the activation of oxidative stress pathways, largely due to their extremely small size and high surface reactivity. Furthermore, microorganisms modulate their energy metabolism in response to stress, while microalgae and fungi may additionally activate apoptosis-related pathways (Hwang et al., 2012; Radhakrishnan et al., 2018; Shen et al., 2020; Ghariani et al., 2025). Similar to other pollutants, long-term exposure to nanomaterials can enhance the development of new traits in microbial communities, including antimicrobial resistance.

## 5 Effects of microplastics on microorganisms

In recent years, plastic pollution has been recognized as one of the major sources of anthropogenic environmental contamination, causing significant harm to both ecosystems and human health (Rhind, 2009; Zhang et al., 2021; Liu L. et al., 2022). In 2020, global plastic production and use totaled 435 million tones, and these amounts are expected to increase by 70% in 2040 (OECD, 2024). The term “microplastics” was first introduced by Thompson et al. (2004). Microplastics are plastic particles such as microbeads, capsules, fibers, or pellets, with sizes ranging from 1 nm to less than 5 mm (Peñalver et al., 2020). However, in the literature, the terms “microplastics” and “nanoplastics” are often used interchangeably (Zhao X. et al., 2023). Thus, in this review, both terms have been considered in the literature analysis, with nanosized plastics included under the definition of microplastics. Microplastics (MPs) can be intentionally manufactured, as in cosmetic products and detergents, or can originate from the degradation of larger plastic materials, including mechanical, chemical, and biological degradation (Peñalver et al., 2020; Stabnikova et al., 2021; Zhao X. et al., 2023). A growing body of research has confirmed the widespread presence of MPs pollution across diverse environments worldwide, including food, bottled and tap water, wastewater, oceans, rivers, agricultural soils, and even the atmosphere (Meng et al., 2025). The most frequently identified plastic polymers in environmental samples include polyethylene (PE), polyvinyl chloride (PVC), polypropylene (PP), polyethylene terephthalate (PET), and polystyrene (PS) (Stabnikova et al., 2021; Zhao X. et al., 2023). Recently, an increasing number of studies have focused on the impact of microplastics exposure on the metabolic profiles of various hosts including animals, plants, humans, and microorganisms (Liu L. et al., 2022; Zhao X. et al., 2023; Meng et al., 2025; Su et al., 2025). Molecular mechanisms related to toxicity and the effects of MPs exposure on the monocultures of microorganisms such as bacteria, algae, and fungi have been investigated. The studies conducted in recent years are summarized in Table 5.

**TABLE 5:** Effects of microplastics (MPs) on microorganisms.

MPs type	MPs particle size	Concentration	Organism	Observed effects	References
Polylactic acid (PLA)	*Nd	100 mg/L	*Bacillus amyloliquefaciens*	Oxidative damage, growth inhibition	Li R. et al., 2023
Polystyrene (PS)	1,040 nm	0–100 mg/L	*Escherichia coli, Acinetobacter sp.*	Decreased cell growth, decreased cell membrane integrity, increased biofilm formation	Kim et al., 2025
Polystyrene (PS)	20 nm–1 mm	0.1–10 mg/L	*Streptomyces coelicolor*	Excessive ROS generation, membrane damage and cell death	Liu et al., 2021
Polystyrene (PS)	30–200 nm	4–32 mg/L	*E. coli*	Increase in ROS, DNA damage growth inhibition	Ning et al., 2022
Polystyrene (PS)	50–55 nm, Amine-modified 1 μm PS bead	80 mg/L	*Halomonas alkaliphila*	Enhanced ROS generation	Sun et al., 2018
Polyethylene (PE), polyamide (PA), polylactic acid (PLA), and polybutylene succinate (PBS)	53–77 μm	10–1,000 mg/L	*Chlorella vulgaris*	Growth inhibition, oxidative damage, EPS production	Su et al., 2022
Polystyrene (PS)	80 nm	5–50 mg/L	*Chlorella pyrenoidosa*	Growth and photosynthetic activity inhibition, ROS generation, membrane damage, induction of apoptosis, alteration in the aminoacyl-tRNA synthetase gene expression levels	Yang W. et al., 2021
Polystyrene (PS)	5 μm	10–100 mg/L	*Scenedesmus obliquus*	Growth inhibition, decreased photosynthetic activity, increased membrane permeability, oxidative stress	Wang et al., 2023
Polyethylene (PE)	50 nm	50 mg/L	*Isochrysis galbana*	Growth inhibition, excessive ROS generation, reduction in chlorophyll *a*, and carotenoid content, alterations in the expression levels of the genes involved in amino acid, fatty acid, and purine metabolism	Xiao et al., 2023b
Polyethylene, polypropylene, and polyvinyl chloride	<100 μm	5–500 mg/L	*Acutodesmus obliquus*	Growth inhibition, decreased photosynthetic activity, changes in protein and carbohydrate content	Ansari et al., 2021
Polystyrene (PS)	80 nm–1 μm	1–20 mg/L	*Heterosigma akashiwo*	Growth inhibition, upregulation of the genes involved in amino acid and energy metabolism	Sheng et al., 2023
Polyethylene (PE), polypropylene (PP), polystyrene (PS), polyvinyl chloride (PVC), polyethylene terephthalate (PET)	200–600 μm	50–100 mg/L	*Scenedesmus sp, Pseudomonas putida, Saccharomyces cerevisiae*	Growth inhibition	Miloloža et al., 2022
Polystyrene (PS)	0.05–1 μm	20–500 mg/L	*S. cerevisiae*	Enhanced ROS generation, growth inhibition, morphological abnormalities	Tang et al., 2025
Polyethylene terephthalate (PET)	56 nm	2–5 mg/mL	*S. cerevisiae*	Oxidative stress, apoptosis and autophagy induction	Kaluç et al., 2024

*Nd, not determined.

Apart from their direct impacts on microorganisms such as toxicity, MPs can also notably influence microorganisms by altering their structural integrity and population dynamics. The majority of research on microbial interactions with microplastics has focused on analyzing microbial community structures (Zettler et al., 2013; Kirstein et al., 2016; Wright et al., 2021; Zhao X. et al., 2023; Messer et al., 2024; Kim et al., 2025). Microplastics impact microorganisms by modifying the structure and function of microbial communities and influencing how they spread (Zhao X. et al., 2023). The surface of MPs provides a special habitat for a variety of microorganisms (Zhao X. et al., 2023). These microorganisms have been observed to colonize the surface of MPs in aquatic environments, which has been shown to create a unique ecosystem, referred to as a “plastisphere” (Zettler et al., 2013; Jacquin et al., 2019). Due to their longevity and high durability, MPs can be transported by wind and ocean currents, thereby facilitating the transfer of microbial communities across various environments (Franzellitti et al., 2019). Zettler et al. (2013) employed next-generation sequencing and SEM to characterize microbial communities associated with plastic surfaces. Their findings suggest that MPs may act as vectors for the transport of pathogenic organisms (Zettler et al., 2013), raising significant environmental and public health concerns due to the potential spread of harmful microbes across ecosystems (Franzellitti et al., 2019).

**FIGURE 2:**
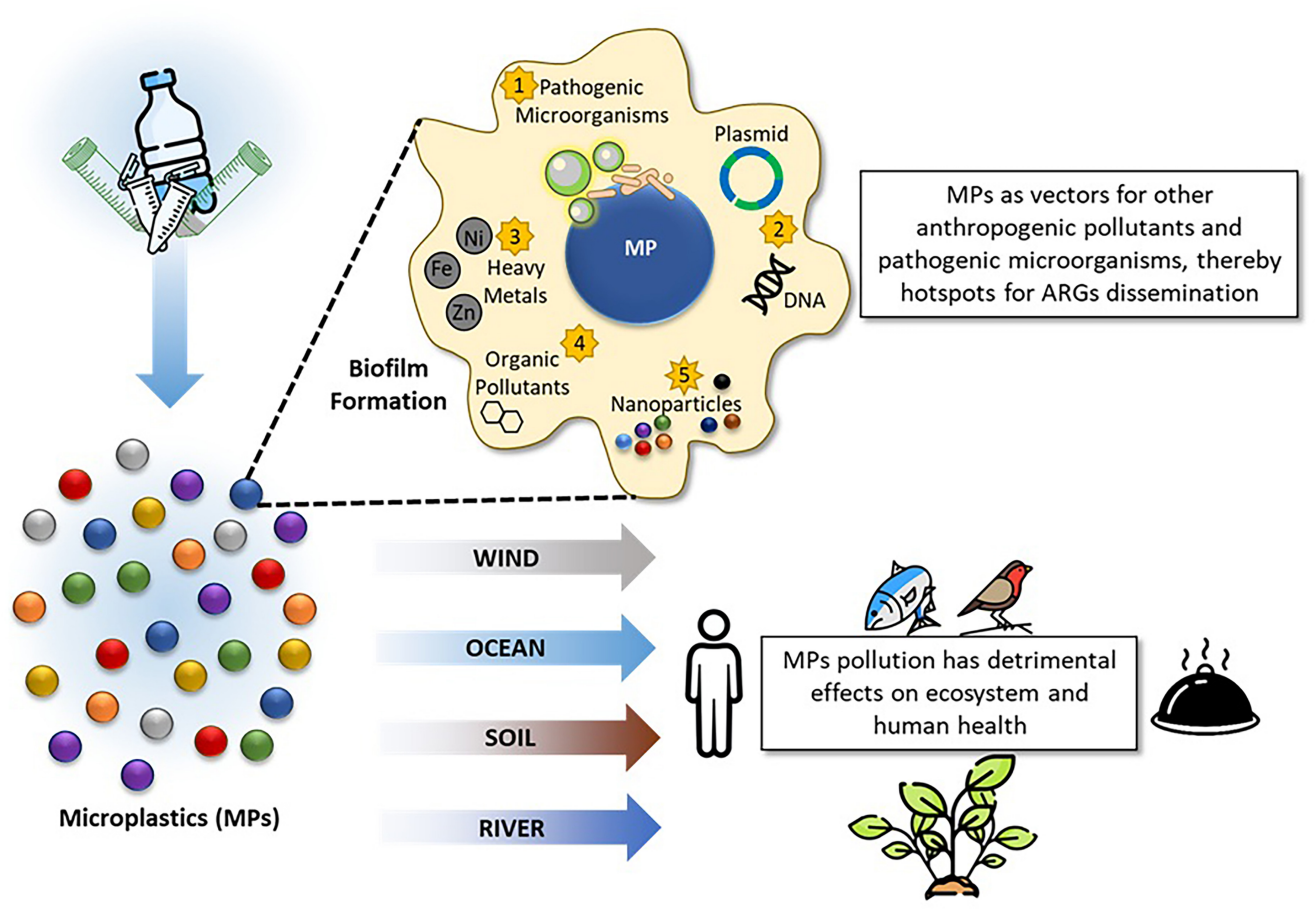
Microplastics (MPs) act as vectors for the propagation of pollutants and pathogenic microorganisms. This promotes horizontal gene transfer and the spread of antibiotic resistance genes (ARGs). Consequently, microplastics act as integrated hubs that amplify the dissemination of resistance, thereby posing risks to the environment and human health.

In addition to microbial communities, MPs can also adsorb other pollutants and act as vectors, facilitating their distribution throughout the environment (Figure 2; Zhao X. et al., 2023; Balta et al., 2025). Due to their hydrophobic nature, extensive surface area, MPs readily adsorb heavy metals, persistent organic pollutants, nanomaterials and antibiotics onto their surfaces. The co-transport of contaminants with MPs has been demonstrated to contribute to more widespread and severe environmental pollution (Balta et al., 2025). The interaction of microplastics with anthropogenic pollutants can occur via various mechanisms. These include van der Waals forces, hydrophobic interactions, hydrogen bonding, electrostatic attraction or repulsion, halogen bonding, π–π interactions, and partitioning effects (Tumwesigye et al., 2023).

The co-occurrence of other pollutants exerts environmental pressure on microorganisms, leading to the spread of antimicrobial resistance and cross-resistance to other pollutants, including heavy metals and organic compounds (Dong et al., 2023). This phenomenon also complicates the study of the effects of combined pollutants on microorganisms (Li R. et al., 2023; Yang et al., 2024). Biofilm formation represents a key factor underlying these processes. Studies have shown that MPs act as substrates for microbial colonization, resulting in the formation of biofilms that can affect the environmental fate of both the MPs and their associated pollutants (Zettler et al., 2013; Wright et al., 2021; Messer et al., 2024). Biofilms formed on MPs exhibit highly heterogeneous structures that can support diverse microbial communities, including potentially pathogenic species (Zettler et al., 2013; Kirstein et al., 2016). Biofilms serve as protective barriers and accumulate nutrients, thereby enabling the coordinated degradation of complex substrates (Kirstein et al., 2016). Furthermore, biofilm formation facilitates the horizontal transfer of genes conferring resistance to pollutants and antibiotics between microbial species. Consequently, MPs serve as substrates that can carry and transport ARGs and act as hotspots for gene exchange (Balta et al., 2025). The mechanisms underlying biofilm-enhanced ARG transfer via horizontal gene transfer are discussed in section “3.1 Persistent pollutants promote antibiotic resistance.” Furthermore, numerous studies have highlighted the ability of microorganisms to biodegrade plastics and microplastics, implying their application potential in mitigating microplastic pollution (Wright et al., 2021; Liu et al., 2023; Gao et al., 2024; Shafana Farveen and Narayanan, 2024). However, the investigation of MPs degradation and its molecular mechanisms is beyond the scope of this review.

It is evident that when considering the collective impact of microplastics on microbial systems, a distinction can be made between two main effects. Direct particle-mediated effects refer to the impact of microplastics on the microorganisms themselves, whereas indirect effects are driven by sorbed contaminants and associated biofilm communities. Although direct interactions with plastic particles may affect microbial physiology, increasing evidence indicates that vector-mediated processes, particularly the accumulation of pollutants and the promotion of biofilm formation, are likely the dominant drivers of microbial adaptation, co-selection, and horizontal gene transfer (Zarean et al., 2025; Balta et al., 2025; Tumwesigye et al., 2023). Consequently, microplastics function as integrated hubs that facilitate the dissemination of other anthropogenic pollutants and microbial communities, thereby amplifying the propagation of resistance traits under the cumulative effect of combined environmental pressures (Figure 2).

### 5.1 Microplastics and bacteria

The toxicity of MPs depends on their physicochemical properties, such as size, concentration, and surface characteristics (Yang et al., 2024). As the size of the MPs decreases, their concentration increases, along with their toxic effects (Yang W. et al., 2021; Tang et al., 2025). Li R. et al. (2023) investigated the effect of polylactic acid (PLA) MPs on *Bacillus amyloliquefaciens.* The findings demonstrated that the presence of PLA has a suppressive effect on the growth and reproduction of *B. amyloliquefaciens*, by impairing its enzymatic antioxidant defenses, damaging cell wall components, and interfering with energy metabolism. As a response against the toxicity induced by PLA MPs, *B. amyloliquefaciens* produces spores (Li R. et al., 2023). Zhang J. et al. (2024) investigated the effects of MPs stress on the chlorine-resistant bacterium *Sphingobium yanoikuyae* in a drinking water distribution system. The study highlighted that these bacteria activate biofilm formation and oxidative stress response mechanisms in the presence of microplastics, primarily affecting their resistance to chlorine. This alteration in resistance poses notable risks, as it could lead to the proliferation of antibiotic-resistant bacterial strains in drinking water systems (Zhang J. et al., 2024). Studies conducted with other bacteria such as *E. coli*, *Acinetobacter sp.*, and *Pseudomonas aeruginosa* also supported the finding that MPs exposure can lead to oxidative stress response and the development of biofilms (Ning et al., 2022; Tao et al., 2024; Kim et al., 2025).

### 5.2 Microplastics and microalgae

The majority of research on microplastics toxicity has predominantly concentrated on microalgae. Exposure to MPs leads to oxidative stress, and inhibition of algal growth and photosynthesis (Yang W. et al., 2021; Wang et al., 2023; Xiao et al., 2023b). Studies conducted with various microalgal species such as *Chlorella pyrenoidosa, Scenedesmus obliquus*, and *Acutodesmus obliquus* have shown that upon exposure, microalgae attach to and aggregate on the surface of MPs (Ansari et al., 2021; Su et al., 2022; Wang et al., 2023). Aggregation of cells on MPs has been found to suppress microalgal growth through various mechanisms, including the blockage of cellular pores, disruption of gas exchange, and hindrance of nutrient availability and light through a shading effect (Ansari et al., 2021; Su et al., 2022). It was also shown that exposure to MPs causes membrane damage and increases membrane permeability, leading to excessive ROS generation, DNA damage, and the induction of apoptosis (Yang W. et al., 2021). Studies have also shown that, in response to oxidative damage, the activity of scavenging enzymes such as SOD and CAT increases in diverse microbial species (Yang W. et al., 2021; Sheng et al., 2023; Wang et al., 2023). In addition, microalgae can secrete extracellular polymeric substances (EPS) to protect themselves from MPs. EPS can immobilize toxic pollutants and reduce their harmful effects (Su et al., 2022; Gao et al., 2023). Transcriptomic and metabolomic studies have revealed that, upon exposure to MPs, the expression of genes related to amino acid, purine, fatty acid, and energy metabolism is altered (Yang W. et al., 2021; Gao et al., 2023; Sheng et al., 2023; Xiao et al., 2023b). The regulatory response enables the inhibition of ROS effects, restoration of cell membrane stability, photosynthetic activity and maintenance of osmotic balance (Yang et al., 2024).

### 5.3 Microplastics and fungi

There is limited research on the effects of MPs on fungi, compared to bacteria and microalgae. Miloloža et al. reported that various MPs inhibit the growth of bacteria (*Pseudomonas putida*), microalgae (*Scenedesmus sp.*) and yeast (*S. cerevisiae*). Among the microorganisms tested, the yeast *S. cerevisiae* was the most sensitive (Miloloža et al., 2022). Studies have shown that positively charged MPs accumulate on the surface of yeast cells and are taken up by the cells (Ozbek et al., 2021; Kojima et al., 2023). MPs attached to the cell surface of *S. cerevisiae* hinder nutrient uptake and inhibit cellular growth (Tang et al., 2025). Ozbek et al. (2021) found that nanosized polystyrene latex (PSL) particles exert toxicity on *S. cerevisiae* when they accumulate inside the vacuoles. Tang et al. (2025) showed that exposure to polystyrene causes morphological abnormalities in *S. cerevisiae*, such as swelling and shriveling. They also found that exposure to MPs results in excessive ROS generation, which triggers increased SOD and CAT activity as a cellular response (Tang et al., 2025). Moreover, Kaluç et al. (2024) reported that PET exposure causes oxidative stress in *S. cerevisiae* and alters the expression of the genes related to stress response and autophagy induction, as a protective mechanism. However, prolonged oxidative stress ultimately leads to apoptosis and cell death in *S. cerevisiae* (Kaluç et al., 2024). MPs have also been found to exert toxic effects on other fungal species, such as *Aspergillus niger* and *Trichoderma harzianum.* The peroxidase and cellobiohydrolase activities of *A. niger* were significantly inhibited upon exposure to micro/nanoplastics (Qv et al., 2022). Exposure of *T. harzianum* to low-density polyethylene microparticles resulted in increased cell membrane permeability, ROS overproduction, and increased SOD and CAT activity (Jasińska et al., 2022). Further research including omics strategies is required to understand the molecular mechanisms of microplastics toxicity in fungi.

Similar to other pollutants, MPs toxicity induces oxidative stress and membrane impairment in microorganisms, thereby leading to growth inhibition. In response, microorganisms activate protective cellular mechanisms, including oxidative stress responses and changes in energy metabolism, while microalgae and fungi may additionally induce apoptosis. However, as discussed in the previous section, MPs act as vectors for the dissemination of other pollutants and ARGs, thereby posing significant risks to ecosystems and human health. Accordingly, further research is required to elucidate the mechanisms underlying microbial responses and resistance.

## 6 The use of evolutionary engineering to understand the molecular mechanisms of microbial response and resistance to anthropogenic pollutants

**FIGURE 3:**
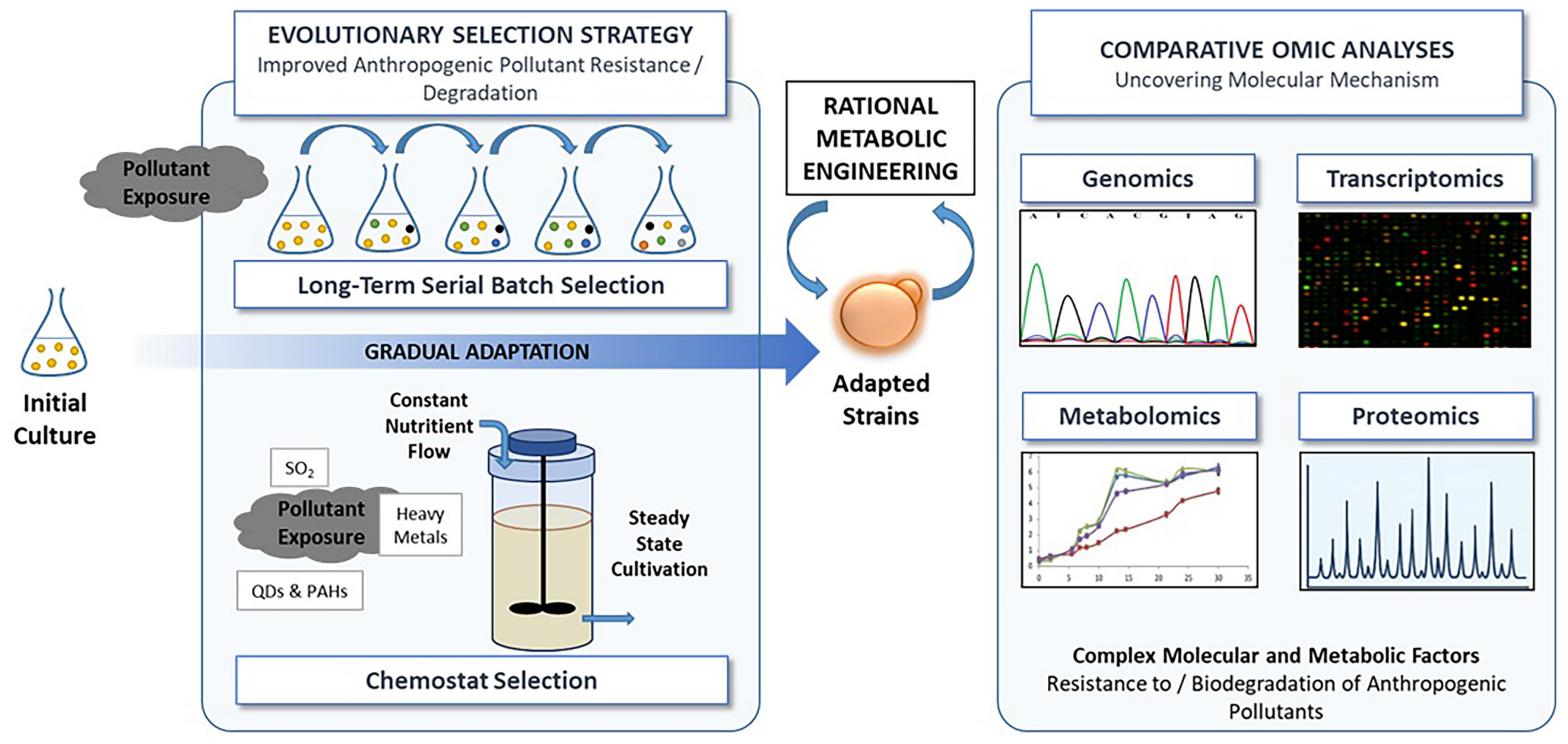
Evolutionary engineering as a potent strategy for investigating microbial responses to anthropogenic stressors. Selective pressure is applied during long-term cultivation (e.g., serial batch or chemostat) to enrich fitter variants that can tolerate anthropogenic stressors. The evolved populations/strains are then analyzed using omics approaches to identify the complex molecular mechanisms underlying stress response and resistance.

Evolutionary engineering, also known as adaptive laboratory evolution (ALE), can also be used as a powerful strategy to investigate the complex molecular basis of the microbial response and resistance to anthropogenic stressors. Evolutionary engineering is an inverse metabolic engineering strategy that utilizes the principles of natural evolution under controlled laboratory conditions. In this approach, a selective pressure that favors the desired phenotype is applied during systematic, long-term (serial batch or chemostat) cultivations (Sauer, 2001; Çakar et al., 2012). Subsequently, the fitter variants obtained from the final populations of selection are analyzed using omics techniques, to uncover the molecular mechanisms underlying stress response and resistance (Çakar, 2009; Mavrommati et al., 2022). However, evolutionary engineering is usually constrained by simplified laboratory selection conditions that may not fully reflect the complexity and fluctuations of the real-world environment or multifactorial stress conditions. Thus, the adaptive trajectories can be limited by mutation supply, population dynamics, and random evolutionary routes which may lead to trade-offs or genomic instability (Dragosits and Mattanovich, 2013; Conrad et al., 2011; LaCroix et al., 2017). On the other hand, some limitations can be mitigated through the use of process-relevant, multistressor selection strategies, automated continuous cultivation and integration of evolutionary engineering with rational metabolic engineering (Topaloğlu et al., 2023). Evolutionary engineering enables researchers to model environmental conditions under controlled conditions. By the long-term application of anthropogenic stressors to microbial cultures during evolutionary engineering, the microbial responses and acquired resistance mechanisms against these stressors can be better understood. A schematic overview of evolutionary engineering is presented in Figure 3. Additionally, evolutionary engineering research conducted using eukaryotic model microorganisms such as the yeast *S. cerevisiae* may help elucidate the long-term effects of anthropogenic pollutants on eukaryotic organisms, including humans. An overview of evolutionary engineering studies for increased microbial resistance to diverse anthropogenic pollutants is shown in Table 6. The majority of the evolutionary engineering studies involving anthropogenic pollutants have focused on heavy metals. Yeast strains that are resistant to diverse heavy metals, e.g., iron (Fe), cobalt (Co), silver (Ag), and nickel (Ni), have been successfully developed using evolutionary engineering strategies. The findings of these studies revealed shared oxidative stress responses that significantly enhance survival of *S. cerevisiae* in response to diverse metal stresses (Çakar et al., 2009; Alkim et al., 2013; Küçükgöze et al., 2013; Balaban et al., 2020; Terzioğlu et al., 2020). Studies on microalgae have also revealed that prolonged exposure to heavy metals during evolutionary engineering selection leads to increased oxidative stress tolerance, enhanced photosynthetic activity (Yu et al., 2020; Marchetto et al., 2025), and cross-resistance to other metals (Xu et al., 2018). Zhang et al. (2019) conducted an ALE study on *E. coli* exposed to heavy atmospheric pollution consisting of metals and PAHs. Omics and physiological analyses revealed that long-term exposure of *E. coli* to diesel exhaust atmosphere with a PM_2.5_ concentration of 613 μg/m^3^ (DEA613) as a multifactorial stress led to the development of resistance through several mechanisms: alterations in cell wall structure and amino acid homeostasis, reduced membrane permeability, and increased exopolysaccharide production (Zhang et al., 2019). Similarly, molecular mechanisms of resistance to another major air pollutant, sulfur dioxide, were investigated in the yeast *S. cerevisiae*, using evolutionary engineering. The resulting evolved strain had mutations in genes related to sulfur dioxide transport, autophagy, and vacuolar protein sorting (Kısakesen et al., 2025). Another study conducted by Strtak et al. (2017) focused on resistance to quantum dot (QD) nanomaterials. The results revealed that *S. cerevisiae* cells adapted to prolonged QD exposure and developed resistance. Whole-genome sequencing results indicated that a mutation in the ubiquitin ligase gene may be associated with the observed resistance to QDs (Strtak et al., 2017). Furthermore, evolutionary engineering has also been applied for the biodegradation of organic pollutants and microplastics (Wang et al., 2024a; Werner et al., 2025). Discovering novel pathways for biodegradation requires extensive knowledge of microbial metabolism. Through evolutionary engineering, microorganisms can be adapted or optimized to degrade anthropogenic pollutants. In addition, comparative omics analyses of the evolved microbial strains and their reference strains can help understand the complex molecular and metabolic factors that enable the biodegradation of anthropogenic pollutants.

**TABLE 6:** Evolutionary engineering or adaptive laboratory evolution (ALE) studies for increased microbial resistance against diverse anthropogenic pollutants.

Anthropogenic pollutant	Evolutionary engineering /ALE strategy	OMICs method	Organism	Outcome	References
Iron (Fe)	Gradually increasing FeCl_2_ concentration (5–15 mM), 15 passages in serial batch cultures	Transcriptomic, genomic	*Saccharomyces cerevisiae*	Increased iron resistance and oxidative stress response, changes in energy and storage metabolism, cross-resistance to chromium, nickel, and cobalt.	Balaban et al., 2020
Silver (Ag)	Gradually increasing AgNO_3_ concentration (5 μM–0.25 mM), 29 passages in serial batch cultures	Transcriptomic, genomic	*S. cerevisiae*	Increased silver resistance and oxidative stress response, alteration in cell wall/membrane, endocytosis, vesicular transport activities, cross-resistance to copper	Terzioğlu et al., 2020
Cobalt (Co)	Gradually increasing CoCl_2_ concentration (0.5–8 mM), 25 passages in serial batch cultures	Transcriptomic	*S. cerevisiae*	Increased cobalt resistance, activation of iron regulon, cross-resistance to nickel, zinc, and manganese	Çakar et al., 2009; Alkim et al., 2013
Nickel (Ni)	Gradually increasing NiCl_2_ concentration (0.05–5.3 mM), 94 passages (about 376 generations) in serial batch cultures	Transcriptomic	*S. cerevisiae*	Increased nickel resistance, upregulation of genes related to iron regulon, oxidative damage, general stress response, cross-resistance to iron, cobalt, and zinc	Küçükgöze et al., 2013
Cadmium (Cd)	Constant exposure to 50 μM CdCl_2_, approximately 200 generations in serial batch cultures	Transcriptomic, genomic	*Chlamydomonas reinhardtii*	Increased cadmium tolerance, mutation in genes involved in photosynthesis, glutathione metabolism, and calcium transport	Yu et al., 2020
Cadmium (Cd)	Gradually increasing CdSO_4_ concentration (4.6–9.0 μM), 128 passages (802 days) in serial batch cultures	Genomic	*Synechocystis sp.*	Increased cadmium resistance, a mutation related to the drug efflux system directly contributing to cadmium resistance, cross-resistance to zinc and cobalt	Xu et al., 2018
Platinum (Pt)	Constant exposure to 62.5 μM PtCl_4_, 8 passages (30 days) in serial batch cultures	Transcriptomic, genomic	*Cupriavidus metallidurans*	Increased resistance to platinum, upregulation of the genes related to cytochrome c and lytic transglycosylase, downregulation of the genes related to pili and flagella	Ali M. M. et al., 2019
Quantum dots (QDs) nanoparticles	Constant exposure to 500 nM QDs, 160 generations (24 days) in serial batch cultures	Genomic	*S. cerevisiae*	Increased resistance to QDs, mutation in the ubiquitin ligase gene *bul1*	Strtak et al., 2017
Atmospheric pollution containing metals and PAHs	Constant exposure to diesel exhaust atmosphere with a PM_2.5_ concentration of 613 μg/m^3^ (DEA613), 56 passages (390 generations) in serial batch cultures	Transcriptomic, genomic	*E. coli*	Mutation in a gene encoding RNA polymerase, altered expression of genes involved in cell membrane/cell wall structure, amino acid and fatty acid metabolism	Zhang et al., 2019
Poly (ethylene terephthalate) (PET)	Constant exposure to 1.66 g/L terephthalate, approximately 199–540 generations (31–82 passages) in an automated ALE system	Genomic	*Pseudomonas putida*	Conversion of PET, mutations in global regulators and catabolic genes	Werner et al., 2025
Phenol	Gradually increasing phenol concentration (200–500 mg/L), 210 days (70 cycles) in batch cultures	Nd	*Dunaliella salina*	Phenol degradation	Wang et al., 2024a
Sulfur dioxide (sodium metabisulfite, K_2_S_2_O_5_)	Gradually increasing K_2_S_2_O_5_ concentration (0.4–1.1 mM), 16 passages in batch cultures	Transcriptomic, genomic	*S. cerevisiae*	Increased resistance to K_2_S_2_O_5_, differential expression of genes related to transport and carbohydrate metabolism, mutations in genes related to SO_2_ transport, autophagy and vacuolar protein sorting, cross-resistance to oxidative, heat and freeze-thaw stress	Kısakesen et al.,2025

## 7 Conclusion

**FIGURE 4:**
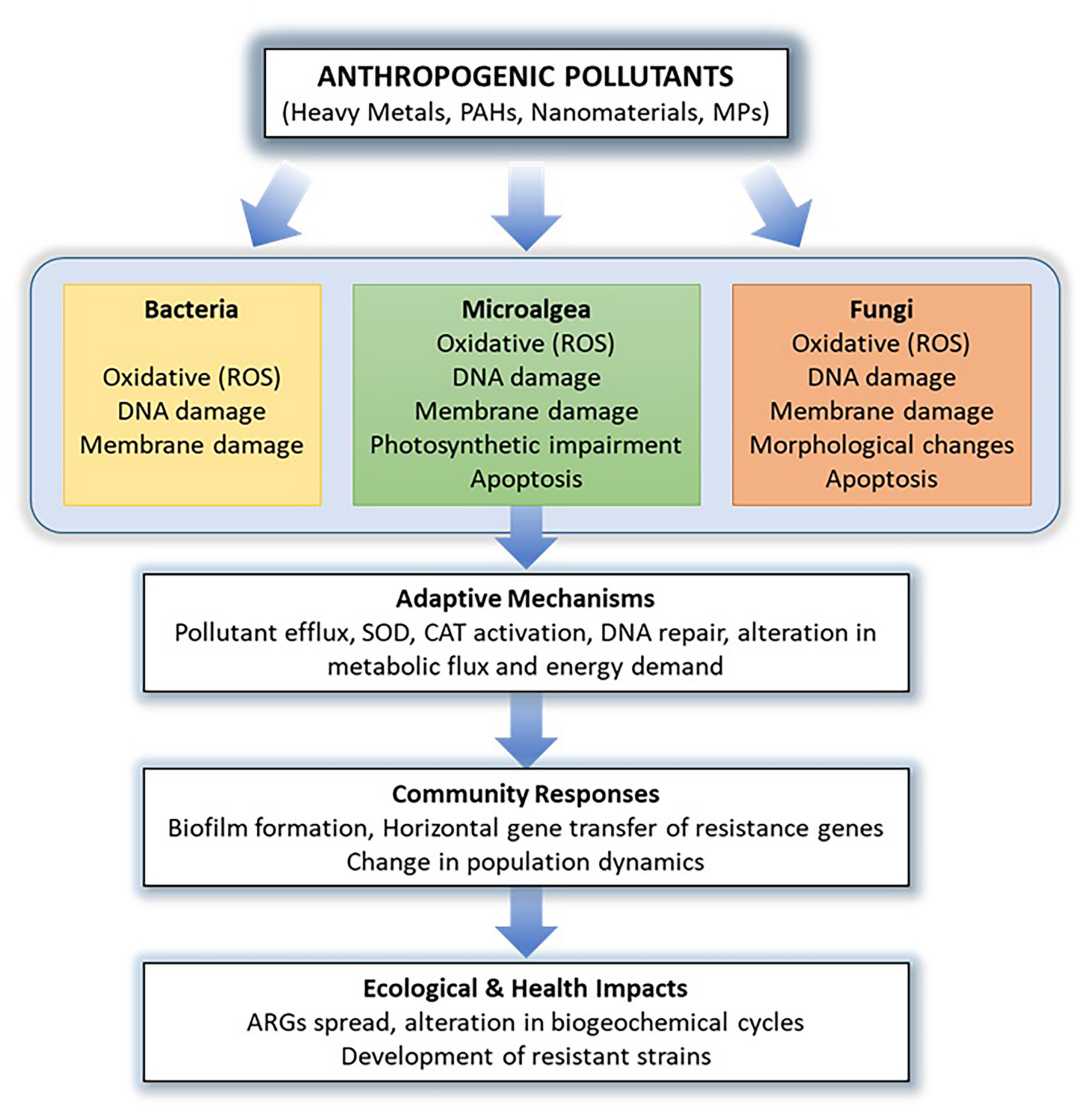
Anthropogenic pollution stress induces stress response and resistance mechanisms in microorganisms, which drive community level adaptive responses, ultimately contributing to resistance development and broader ecological impacts.

As the ecological implications of anthropogenic pollutants expand, it is imperative to understand microbial response and resistance mechanisms to these stressors. This review aims to integrate a conceptual framework of microbial responses and resistance across different classes of pollutants. Despite the chemical diversity of these stressors, microbial responses converge on a limited set of core stress-response mechanisms. Conserved mechanistic pathways include the induction of oxidative stress, activation of adaptive systems such as efflux pumps and detoxification processes, and alterations in cellular energy demand. Divergence in responses primarily occurs at the level of organism-specific physiology; for example, photosynthetic impairment in microalgae and morphological adaptations in fungi and physicochemical nature of pollutants. As summarized in Figure 4, microbial responses to pollutants can be conceptualized as a linkage between anthropogenic pollutants stress to ecological outcomes including dissemination of antibiotic resistance genes (ARGs), spread of more resistant strains and, alteration of biogeochemical cycles.

Through an omics-based exploration of adaptive mechanisms, the effects of anthropogenic pollutants on microorganisms can be investigated at a molecular level. Future research should employ integrative, community-level and multi-omics approaches to better understand these interactions. Furthermore, emerging studies leveraging adaptive laboratory evolution or evolutionary engineering strategies that simulate environmentally relevant conditions provide a predictive framework for understanding microbial evolution under human-made stressors. Integrating these approaches not only deepens our ecological understanding but also supports the development of pollutant-tolerant strains for bioremediation, bio-monitoring, and synthetic biology applications.
